# Discovery of putative inhibitors against main drivers of SARS-CoV-2 infection: Insight from quantum mechanical evaluation and molecular modeling

**DOI:** 10.3389/fchem.2022.964446

**Published:** 2022-10-11

**Authors:** Toheeb A. Balogun, Onyeka S. Chukwudozie, Uchechukwu C. Ogbodo, Idris O. Junaid, Olugbodi A. Sunday, Oluwasegun M. Ige, Abdullahi T. Aborode, Abiola D. Akintayo, Emmanuel A. Oluwarotimi, Isaac O. Oluwafemi, Oluwatosin A. Saibu, Prosper Chuckwuemaka, Damilola A. Omoboyowa, Abdullahi O. Alausa, Nkechi H. Atasie, Ayooluwa Ilesanmi, Gbenga Dairo, Zainab A. Tiamiyu, Gaber E. Batiha, Afrah Fahad Alkhuriji, Wafa Abdullah I. Al-Megrin, Michel De Waard, Jean-Marc Sabatier

**Affiliations:** ^1^ Department of Biochemistry, Adekunle Ajasin University, Akungba-Akoko, Nigeria; ^2^ Department of Biological Sciences, University of California, San Diego, San Diego, CA, United States; ^3^ Department of Applied Biochemistry, Nnamdi Azikiwe University, Awka, Nigeria; ^4^ Department of Chemistry and Chemical Biology, Stevens Institute of Technology, Hoboken, NJ, United States; ^5^ Department of Environmental Toxicology, Universitat Duisburg-Essen, Essen, Germany; ^6^ Department of Marine Biological Resources, Ghent University, Ghent, Belgium; ^7^ Department of Chemistry, Mississippi State University, Starkville, MS, United States; ^8^ Department of Chemistry, University of Texas at Dallas, Richardson, TX, United States; ^9^ Department of Chemistry, Missouri University of Science and Technology, Rolla, MO, United States; ^10^ Department of Chemistry, Adekunle Ajasin University, Akungba-Akoko, Nigeria; ^11^ Department of Biotechnology, Federal University of Technology Akure, Akure, Nigeria; ^12^ Department of Biochemistry, Ladoke Akintola University, Ogbomoso, Nigeria; ^13^ Clinical Pharmacy Department, Nigeria Correctional Service, Enugu Custodial Centre, Enugu, Nigeria; ^14^ Department of Chemistry, Mississipi University for Women Columbus, Columbus, United States; ^15^ Department of Biological Sciences, Western Illinois University, Macomb, IL, United States; ^16^ Department of Biochemistry and Molecular Biology, Federal University Dutsin-ma, Dutsin-Ma, Nigeria; ^17^ Department of Pharmacology and Therapeutics, Faculty of Veterinary Medicine, Damanhour University, Damanhour, Egypt; ^18^ Department of Zoology, College of Science, King Saud University, Riyadh, Saudi Arabia; ^19^ Department of Biology, College of Science, Princess Nourah bint Abdulrahman University, Riyadh, Saudi Arabia; ^20^ Smartox Biotechnology, Saint-Egréve, France; ^21^ L‘institut du Thorax, INSERM, CNRS, Université de Nantes, Nantes, France; ^22^ LabEx Ion Channels, Science and Therapeutics, Université de Nice Sophia-Antipolis, Valbonne, France; ^23^ Institut de Neurophysiopathologie (INP), Faculté des Sciences Médicales et Paramédicales, Aix-Marseille Université, CNRS UMR 7051, Marseille, France

**Keywords:** SARS-CoV-2, COVID-19, 3CLpro, PLpro, RNA-dependent RNA polymerase (RdRp), molecular modeling, glycoprotein

## Abstract

SARS-CoV-2 triggered a worldwide medical crisis, affecting the world’s social, emotional, physical, and economic equilibrium. However, treatment choices and targets for finding a solution to COVID-19’s threat are becoming limited. A viable approach to combating the threat of COVID-19 is by unraveling newer pharmacological and therapeutic targets pertinent in the viral survival and adaptive mechanisms within the host biological milieu which in turn provides the opportunity to discover promising inhibitors against COVID-19. Therefore, using high-throughput virtual screening, manually curated compounds library from some medicinal plants were screened against four main drivers of SARS-CoV-2 (spike glycoprotein, PLpro, 3CLpro, and RdRp). In addition, molecular docking, Prime MM/GBSA (molecular mechanics/generalized Born surface area) analysis, molecular dynamics (MD) simulation, and drug-likeness screening were performed to identify potential phytodrugs candidates for COVID-19 treatment. In support of these approaches, we used a series of computational modeling approaches to develop therapeutic agents against COVID-19. Out of the screened compounds against the selected SARS-CoV-2 therapeutic targets, only compounds with no violations of Lipinski’s rule of five and high binding affinity were considered as potential anti-COVID-19 drugs. However, lonchocarpol A, diplacol, and broussonol E (lead compounds) were recorded as the best compounds that satisfied this requirement, and they demonstrated their highest binding affinity against 3CLpro. Therefore, the 3CLpro target and the three lead compounds were selected for further analysis. Through protein–ligand mapping and interaction profiling, the three lead compounds formed essential interactions such as hydrogen bonds and hydrophobic interactions with amino acid residues at the binding pocket of 3CLpro. The key amino acid residues at the 3CLpro active site participating in the hydrophobic and polar inter/intra molecular interaction were TYR54, PRO52, CYS44, MET49, MET165, CYS145, HIS41, THR26, THR25, GLN189, and THR190. The compounds demonstrated stable protein–ligand complexes in the active site of the target (3CLpro) over a 100 ns simulation period with stable protein–ligand trajectories. Drug-likeness screening shows that the compounds are druggable molecules, and the toxicity descriptors established that the compounds demonstrated a good biosafety profile. Furthermore, the compounds were chemically reactive with promising molecular electron potential properties. Collectively, we propose that the discovered lead compounds may open the way for establishing phytodrugs to manage COVID-19 pandemics and new chemical libraries to prevent COVID-19 entry into the host based on the findings of this computational investigation.

## 1 Introduction

With the emergence of different variations of the coronavirus disease 2019 (COVID-19), such as alpha, delta, and omicron, COVID-19 remains a global challenge to health and the economy due to the unexpected emergence of severe acute respiratory syndrome coronavirus-2 (SARS-CoV-2). COVID-19 is an agile respiratory disease caused by a novel coronavirus first reported in Wuhan, China, in December 2019 and declared a global pandemic by the World Health Organization (WHO) in March 2020 (Chan et al., 2020). Coronaviruses are positive sensed, linear single-stranded RNA viruses which comprise nucleoproteins (N), envelope proteins (E), matrix proteins (M), spike proteins (S), and many non-structural proteins (Luk et al., 2019). The length of the RNA genomes ranges from 26–32 kb, which contains 12 open reading frames. A structural analysis of the SARS-CoV-2 genomes using biophysical and modeling techniques reveals that two polyproteins divided into 15 or 16 non-structural proteins make up the first two-thirds of the coronavirus genome (Xu et al., 2020). A phylogenetic analysis shows that the remaining ORFS contains the genetic makeup of four important structural proteins: envelop (E), membrane (M), nucleocapsid (N), and spike (S) proteins. These proteins played a significant role in the virus survival and replication and have received intense interest from several investigators to develop inhibitors of SARS-CoV-2 (Adedeji et al., 2012; Yu et al., 2012; Jia et al., 2019). Notably, COVID-19 has been associated with an effect on multiple vital organs and the central nervous system and can result in respiratory problems with fatal consequences (Seah and Agrawal, 2020). Previous reports indicated that SAR-CoV-2 therapeutic targets include receptor binding of glycosylated spike (S) protein, which mediates host cell receptor recognition and host cell entry and induces host immune responses, and non-structural proteins such as RNA-dependent RNA polymerase (RdRp), CoV main protease [Mopar; also known as 3-chymotrypsin–like protease (3CLpro)], and papain-like protease (PLpro) (Liu et al., 2020). Theoretically, drugs competing with RBD for receptor binding sites can inhibit viral entry and replication. Furthermore, the non-structural proteins (PLpro, 3CLpro, and RdRp) play a vital role in proteolysis and viral polyprotein processing. Thus, NSPs had recently emerged as an essential therapeutic biomarker for the design of COVID-19 drug candidates (Chen et al., 2020).

The therapeutic management of COVID-19 involves two target selection approaches. One of the methods is boosting the human immune system or human cells using an attenuated vaccine, whereas the second technique involves inhibiting molecular targets by small molecule inhibitors. Regarding the human immune system, the innate immune system plays a key role in disrupting coronavirus replication and its entry. As expected, the interferon helps to enhance the immune response to the virus (Omrani et al., 2014). One of the most effective ways to halt viral replication and entry is using small molecules to block the signaling pathways of human cells which mediate virus replication. Furthermore, viruses interact with certain receptor proteins on the surface of cells to gain entry into human cells. Notably, RBD of SARS-CoV-2 binds with the human angiotensin-converting enzyme 2 (ACE-2) receptor (Li et al., 2003; Han et al., 2006; Ge et al., 2013).

Scientists have harnessed several strategies for developing novel drugs against COVID-19 (Zumla et al., 2016). The first strategy was to screen existing broad-spectrum anti-viral drugs such as ribavirin and cyclophilin. This approach is advantageous because the pharmacokinetic profile of the anti-viral drugs and their associated side effects have been clearly stated. One of the disadvantages of broad-spectrum anti-viral drugs is their non-specificity, which might, in turn, result in low potency against coronavirus (Chan et al., 2013; de Wilde et al., 2014). The second techniques involve screening molecular databases such as the ZINC database to identify potential anti-coronavirus compounds *via* high-throughput virtual screening. This approach has been used to develop biologically active compounds, lopinavir/ritonavir, as anti-HIV agents (de Wilde et al., 2014). The third approach is based on analyzing genomic datasets to develop new targeted drug candidates from scratch for precision medicine (Dyall et al., 2014). Therapeutic agents developed against coronavirus *via* the third strategy exhibits promising pharmacological potential. However, this approach’s long-term process and expenses are major limiting factors.

Herbal medicine has been an alternative medicine since time immemorial in managing various diseases and may be an important source of anti-coronavirus agents (Ling, 2020). An earlier systematic study in 2003 shows that patients infected with SARS-CoV-2 treated with traditional Chinese medicine (TCM) were reported to have short time hospitalization, reduced steroid side effects, and improvements from the viral symptoms (World Health Organization, 2004). Therefore, a significant amount of research has focused on developing therapeutic agents against coronavirus from TCM, ethnobotanical herbs, and dietary supplements (Dudani and Saraogi, 2020; Vellingiri et al., 2020). *In vivo*, *in vitro*, and *in silico* studies have revealed the antiviral potential of numerous bioactive compounds against coronavirus. Some phytocompounds and sources include glycyrrhizin isolated from *Glycyrrhiza glabra* L. (licorice); tetra-O-galloyl-β-D-glucose (TGG) and luteolin, isolated from *Rhus chinensis* Mill. and *Veronica linariifolia* Pall. ex Link; and aurantiamide acetate derived from *Artemisia annua* L. plant. Several plants such as *Sanguisorba officinalis* L., *Stephania tetrandra* S. Moore, and *Strobilanthes cusia* (Nees) Kuntze have been reported for their antiviral potential toward RNA and protein synthesis of the coronavirus (Wu et al., 2004; Wang et al., 2007; Kim et al., 2010). In this study, manually curated compounds library from some medicinal plants were screened against four main targetable drivers of SARS-CoV-2 (spike glycoprotein, PLpro, 3CLpro, and RdRp) using high-throughput virtual screening. Only compounds with no violations of the Lipinski’s rule of five with at least a binding energy of −5.0 kcal/mol against the four targets were considered potential drug candidates with therapeutic effects against COVID-19. Herein, lonchocarpol A, diplacol, and broussonol E (lead compounds) were recorded as the best compounds that satisfied this requirement and demonstrated their highest binding affinity against 3CLpro. Therefore, the 3CLpro target and the three lead compounds were selected for further analysis, such as molecular dynamics simulation and quantum mechanical evaluation. Overall, this study serves as a benchmark for developing the discovered three lead compounds as COVID-19 therapeutic agents.

## 2 Materials and methods

### 2.1 Quantum mechanical calculation

#### 2.1.1 Molecular docking studies

Theoretical approaches used to compute compounds’ chemical and biological activities have been well documented. The quantum chemical (QM) calculation was executed using the MOPAC 2016 software program (Stewart, 1990). PM7 semi-empirical Hamiltonian incorporating an implicit COSMO solvation model was used to perform the calculation (Klamt and Schuurmann, 1993; Dutra et al., 2013). Notably, geometric pre-optimization of the top four inhibitors was carried out using the molecular mechanics force field (MMFF94) integrated with the Avogadro v 1.2.0 software program (Hanwell et al., 2012), coupled with chemical structure protonation at a pH of 7.4. The pre-optimized geometry serves as a query for QM calculation (Chukwuemeka et al., 2021). A Broyden–Fletcher–Goldfarb–Shanno (BFGS) geometry optimizer was used for structure minimization and optimization at the semi-empirical theory level. The keywords “DIPOLE” and “MULLIK” were used to compute the dipole moments and Mulliken atomic charges, respectively. The time-dependent Hartree Fock (TDHF) was used to calculate the molecular polarizabilities using the “POLAR” keyword incorporated in MOPAC 2016. A visualization tool (Jmol software program) was used to visualize the charge distribution diagram of frontier molecular orbitals (FMOs) and molecular electrostatic potential (MEP) of the docked compounds (Hanson, 2010). A quantum chemical calculation *via* density functional theory (DFT) was used to investigate the physicochemical properties of lead phytocompounds with the best conformer distribution. All the quantum chemical reactivity descriptors were computed from the energies of the highest occupied and lowest unoccupied molecular orbitals (EHOMO–LUMO). The descriptors include the following:

• Energy band gaps (Eg).
• Ionization energy (I).
• Electron affinity (A).
• Chemical hardness (*η*).
• Chemical softness (*δ*).
• Chemical potential (*μ*).
• Electronegativity (*χ*).

#### 2.1.2 Protein and ligand preparation

The crystal structures of the molecular targets RBD of spike glycoprotein (PDB: 6MOJ), 3CLPro (PDB: 6M2N), PLPro (7CJM), and RdRp (7D4F) were obtained from the protein data bank (https://www.rcsb.org/). They were prepared using Schrödinger’s protein preparation wizard (Sastry et al., 2013). Hydrogen bond optimizations, water removal, protein structure correction, and ultimately protein energy minimization using the OPLS_2005 force field were carried out during the preparation. The position of the co-crystallized ligands for each target was used to define the protein binding pocket for receptor grid generation. Subsequently, the 3D structure of 1,000 compounds consisting of the substructure of the co-crystallized ligand of the targets was downloaded from the PubChem database (https://pubchem.ncbi.nlm.nih.gov). The structures of the ligands were cleaned, and their geometries were subjected to structural optimization using the default specifications of the LigPrep module incorporated in the Schrodinger suite and utilized for hypothesis generation (Balogun et al., 2021a). The prepared proteins and fully optimized geometry of ligands were used as input for molecular docking.

#### 2.1.3 Molecular docking

High-throughput virtual screening (HTVS) of the prepared phytocompounds library was performed using the HTVS module of Maestro integrated into the Schrodinger suite (Friesner et al., 2004). The HTVS module used the 3D crystallographic structure of the therapeutic targets and fitted the ligands based on their structural conformations. During the virtual screening process, an energy score of −5.0 kcal/mol was set as the threshold to identify potential anti-coronavirus agents. The hits generated, 105 compounds out of 1,000 screened phytocompounds, were subjected to molecular docking by considering the flexibility of the protein using the SP (standard precision) model. The compounds were subjected to XP (extra precision) docking using the GLIDE XP module incorporated in Maestro to achieve further accurate results based on binding affinity and pose. The structural and energy information between the protein–ligand complexes was considered for energetic computation and further stability studies. The 2D interaction profile of protein–ligand complexes was generated using the Ligand Interaction Diagram (LID) in Maestro (Friesner et al., 2004; Balogun et al., 2021b). The reproducibility and reliability of the docking procedure were validated by superimposing and re-docking the co-crystalized ligand structures into the target active site, which generated an RMSD value of 0.76 A (normal range: 0–2 A). This confirms the reliability of the docking protocol.

### 2.2 Binding free energy and contribution energies calculation using MM-GBSA

The XP-screened compounds were further subjected to a Prime MM/GBSA analysis, where their binding energies were computed to investigate the inhibitory potential of the docked compounds against the targets. Based on the number of energy parameters generated by the Prime algorithm, free energy parameters were used to gain mechanistic insight into the biological activity of the compounds. Nonetheless, the ligand strain energy, Coulomb energy, and van der Waals energy were also assessed in filtering the final hit compounds (Genheden and Ryde, 2015; Schrödinger, 2020). The binding free energy and essential amino acid interactions between the protein–ligand complexes were computed using the following equations:

$$\Delta \text{Gbind}\mathrm{=\Delta}\text{E}\mathrm{+\Delta}{\text{G}}_{\text{solv}}\mathrm{+\Delta}\text{GSA}\text{.}$$ (1)

ΔE=E(complex)−E(protein)−E(ligand), (2)

where E(complex), E(protein), and E(ligand) are the minimized energies of the protein–inhibitor complex, protein, and inhibitor, respectively.

ΔGsolv=Gsolv(complex)−Gsolv(protein)−Gsolv(ligand), (3)

where Gsolv(complex), Gsolv(protein), and Gsolv(ligand) are the salvation free energies of the complex, protein, and inhibitor, respectively.

ΔGSA=GSA(complex)−GSA(protein)−GSA(ligand), (4)

where GSA (complex), GSA (protein), and GSA (ligand) are the surface area energies for the complex, protein, and inhibitor, respectively.

### 2.3 Drug likeness and ADMET evaluation

To compute the lead compounds’ physicochemical parameters and pharmacokinetic models, the compounds inputted structures were transformed into their canonical simplified molecular input line entry system (SMILES) form. Therefore, the curated ligand database’s SMILES were uploaded to the admetSAR web server (http://lmmd.ecust.edu.cn/) (Cheng et al., 2012) and SwissADME (Daina et al., 2017). Drug-likeness is a method for determining if a therapeutic agent is appropriate for orally active medications. Lipinski’s rule of five principles are used to compute *in silico* predictions based on parameters such as molecular weight, hydrogen bond donor, and hydrogen bond acceptor (Lipinski et al., 2001).

### 2.4 Molecular dynamics simulation

Molecular dynamics (MD) simulations were conducted to predict the protein’s dynamic motion and stability at the atomistic level with the bounded protein. The DESMOND module integrated into the Schrodinger suite was used to generate protein–ligand topologies and trajectories. The protein–ligand complexes were performed for 100 ns with an OPLS3 force field, using the DESMOND version of Schrödinger (2018). The solvation box was designed as the shape of the rhombic dodecahedron type and solvated using the TIP3P (transferable intermolecular potential 3 point) and an orthorhombic box (10 Å × 10 Å × 10 Å buffer) water model. Na^+^ and Cl^−^ ions in 0.15 mM concentration were added to neutralize the charge of the systems during simulation. The system minimization tool in the Desmond–Maestro interface was used for energy minimization of the complete system under default parameters of 1.0 kcal/mol/Å, convergence threshold, and maximum iterations of 2,000. Furthermore, the system was calibrated with the constant temperature (300 K) and pressure (1 bar) *via* Berendsen thermostat coupling and default system pressure coupling, respectively. Each of the equilibration steps was carried out for 100 ps. The dynamic simulation of the complex system was performed for 10 ns after all the pre-processing phases. The V-rescale and Parrinello–Rahman methods were used for temperature and pressure coupling, respectively (Parrinello and Rahman, 1981). Leonard-Jones potential and the particle mesh Ewald (PME) method were used to handle van der Waals and long-range electrostatic interactions, respectively (Darden et al., 1993). The complexes underwent a final MD simulation production run for 100 ns. Root-mean-square deviations (RMSD) were computed using the MD trajectory to estimate the variations in protein conformation during the various simulations period, and root-mean-square fluctuation (RMSF) as well as the total number of intermolecular contacts were used for each protein–ligand complex to gain insights into the compound’s inhibitory potential (Pearson, 1986).

## 3 Results and discussion

### 3.1 Frontier molecular orbital analysis and global reactivity descriptors

FMOs such as HOMO and LUMO are essential in demystifying the chemical reactivity at the atomic level and are crucial descriptors for rationalizing various chemical reactions. The reactivity descriptors calculated for lonchocarpol A, broussonol A, diplacol, and dexamethasone are shown in Table 1. HOMO energy denotes the potential of a molecule to easily donate an electron, which also corresponds to the ionization potential of a molecule. In contrast, the electron-withdrawing potential of compounds is referred to as the LUMO energy, which signifies the first empty innermost orbital unfilled by an electron and correlates with a molecule’s electron affinity. The band gap energy is the difference between the HOMO and the LUMO energy and provides information about the compound’s chemical stability at the molecular level. Band gap energy also describes the chemical reactivity of a molecule, deciphering the movement of electrons from the ground state to its excitation state. Furthermore, other parameters (such as chemical hardness, softness, electronegativity, or polarizability) that provide information about compounds’ ionic structure and the electronic configuration can be easily computed *via* HOMO–LUMO energy (Pearson, 1986; Sylaja et al., 2017). For example, a lower energy gap between two frontier molecular orbitals means lower kinetic stability and higher polarizability and reactivity of a molecule, which indicates the softness of the molecule and vice versa.

**TABLE 1:** Calculated quantum reactivity descriptors of top four compounds using the PM7 Hamiltonian method.

SN	Quantum chemical property	Lonchocarpol A	Broussonol E	Diplacol	Dexamethsaone
1	HOMO	−8.776 eV	−8.647 eV	−8.763 eV	−9.964 eV
2	LUMO	−0.501 eV	−1.117 eV	−0.902 eV	−0.501 eV
3	Energy gap (ΔE)	−8.275 eV	−7.530 eV	−7.861 eV	−8.275 eV
4	Ionization potential (I)	8.776 eV	8.647 eV	8.763 eV	8.776 eV
5	Electron affinity (A)	0.501 eV	1.117 eV	0.902 eV	0.501 eV
6	Chemical hardness (*η*)	4.138 eV	3.765 eV	3.931 eV	4.138 eV
7	Chemical softness (*ζ*)	0.242 (eV)^−1^	0.267 (eV)^−1^	0.254 (eV)^−1^	0.242 (eV)^−1^
8	Electronegativity (*χ*)	4.639 eV	4.882 eV	4.833 eV	4.639 eV
9	Chemical potential (*µ*)	−4.634 eV	−4.882 eV	−4.833 eV	−4.634 eV
10	Electrophilicity index (*ω*)	2.595 eV	3.165 eV	2.971 eV	2.595 eV

ΔE = HOMOε−LUMOε, I = −E_HOMO_, A = −E_LUMO_, η = (I − A)/2, ζ = 1/η, χ = (I + A)/2, µ = − (I + A)/2, ω = µ^2^/2η.

**FIGURE 1:**
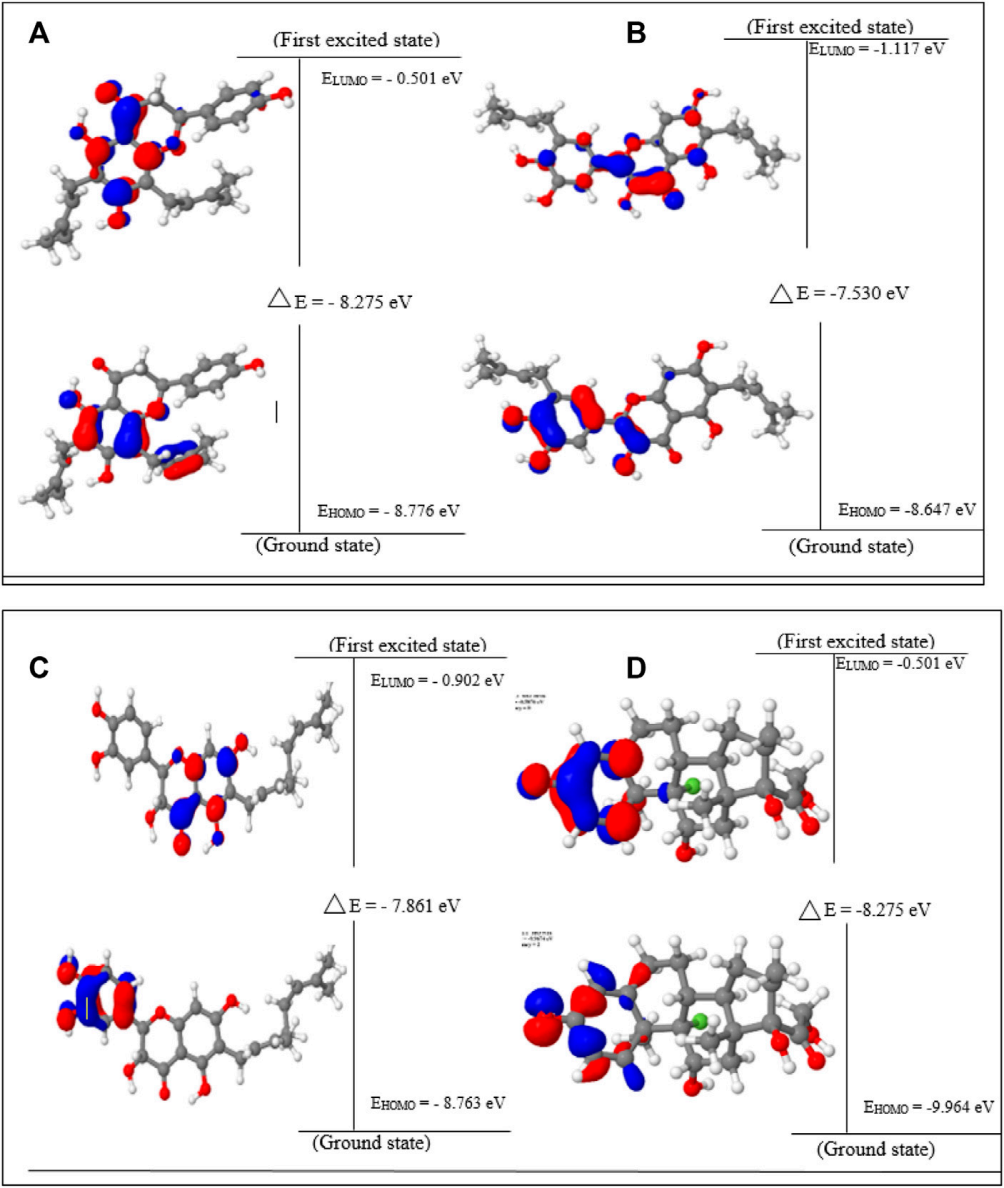
HOMO, LUMO, and band gap energy (∆E) of the top four compounds: **(A)** lonchocarpol A, **(B)** broussonol E, **(C)** diplacol, **(D)** dexamethasone.

Lonchocarpol A has the second highest HOMO energy value (EHOMO = −8.77 eV), denoting the valence electron density distribution for lochocarpol A is more available to be donated, suggesting that lonchocarpol A is the most reactive compound after dexamethasone. Similarly, broussonol E and diplacol recorded a HOMO energy value of −8.647 and −8.763 eV, respectively. Clearly, lonchocarpol A, broussonol E, and diplacol demonstrated an intermolecular charge transfer as they excited from the ground state (S0) to the first excitation state. Interestingly, the LUMO energy is in the following order: lochocarpol A < dexamethasone < diplacol < broussonol E. The LUMO energy suggests that lonchocarpol A and dexamethasone are more susceptible to accepting electronic density because a lower energy molecular orbital will describe the additional electron. The chemical reactivity of a compound is measured using the HOMO–LUMO energy gap (ΔEGap), which represents a lower energy difference (lower energy gap) (Figure 1).

Broussonol E had the lowest energy value of −7.530 eV compared to dexamethasone (−8.275 eV), which implies more chemical reactivity to broussonol E. Lonchocarpol A and dexamethasone showed the same energy gap (−8.275 eV) which is in consistent with their LUMO values. This suggests that both the compounds may share similar chemical reactivity properties and mechanisms of action toward the targets. To fully gain insights into the reactivity and chemical species of the top four compounds with drug-likeness properties, the following parameters were evaluated from the HOMO and LUMO energy: ionization potential, electron affinity, chemical hardness (*η*), chemical softness (*ζ*), electronic chemical potential (*µ*), electrophilicity index (*ω*), and electronegativity (*χ*). The expression for the reactivity parameters, as mentioned earlier, has been described according to Koopman’s theory (Phillips, 1961) and can be calculated by the following mathematical statements:

$$\text{Energy Gap }\Delta \text{E}=HOMO_{\varepsilon }-LUMO_{\varepsilon };$$ (5)

$$\text{Ionization Potential I}\mathrm{=-}E_{HOMO};$$ (6)

$$\text{Electron affinity A}=-E_{LUMO};$$ (7)

$$\text{Chemical hardness }\mathrm{\eta =}\frac{1}{2}\left(\frac{\partial^{2}E}{\partial N^{2}}\right)V=\frac{1}{2}\left(\frac{\partial µ}{\partial N}\right)V=\left(I-A\right)/2;$$ (8)

$$\text{Chemical potential }\mu =\left(\frac{\partial E}{\partial N}\right)V=-\left(I+A\right)/2;$$ (9)

$$\text{Electronegativity }\chi \mathrm{=-}\mu =-\left(\frac{\partial E}{\partial N}\right)V=\left(I+A\right)/2;$$ (10)

$$\text{Softness }\zeta =\frac{1}{\eta };$$ (11)

$$\text{Electrophilicity index }\omega =\frac{µ2}{2\eta }.$$ (12)

**FIGURE 2:**
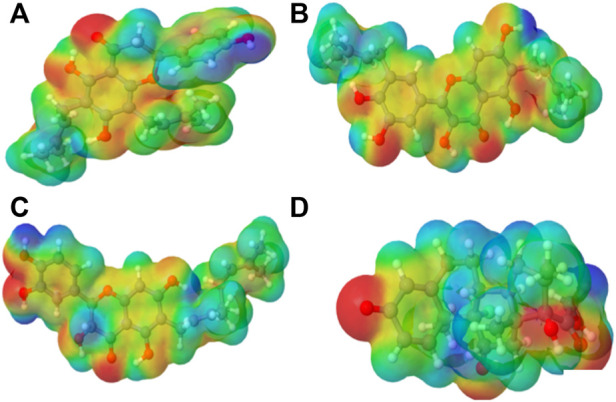
Molecular electrostatic potential (MEP) of the top four compounds: **(A)** lonchocarpol A, **(B)** broussonol E, **(C)** diplacol, **(D)** dexamethasone.

Ionization energy helps to determine the amount of free energy required to remove an electron of an atom from a molecule. Furthermore, electron affinity represents the amount of energy liberated when an atom or molecule is attached to a neutral atom or molecule. Lower ionization potential indicates lower stability or higher reactivity of the compound and its contribution toward analyzing inhibitory potential. Contrarily, electron affinity depicts the high electron-withdrawing ability of a compound. Table 1 shows that lochocarpol A and dexamethasone had the highest chemical stability and electron-withdrawing potential compared to broussonol E and diplacol. This observation is consistent with the gap energy between the HOMO and LUMO FMOs (Figure 2). The softness and hardness properties of compounds contribute to their chemical stability. Although a higher hardness value means a more stable chemical entity, compound’s stability decreases with softness. Pearson’s HSAB theory proposed that a favorable interaction between two compounds occurs when both are hard and soft (Pearson, 1990; Arjunan and Mohan, 2009). It is evident from Table 1 that lonchocarpol A and dexamethasone have chemical hardness values of 4.138 eV, indicating they are the most stable compound, followed by diplacol (3.931 eV) and broussonol E (3.765). The chemical softness of the compounds shows that there is only a subtle difference between the compounds denoting their chemical stability. The ability of a compound to not decompose spontaneously into an element, which denotes its stability, is determined by a higher negative chemical potential. All the compounds demonstrated chemical stability due to their negative value of chemical potential. Electronegativity and electrophilicity are another important set of reactivity descriptors. Lonchocarpol A and dexamethasone have the same electrophilicity index (2.595 eV) and electronegativity values (4.639 eV), which implies their susceptibility to accept electron density and classifies them as promising electrophilic compounds. Broussonol E was recorded as the most electrophilic molecule with an electrophilicity index value of 3.165 eV. Therefore, Table 1 provides appropriate information regarding the chemical reactivity and stability of the studied compounds.

### 3.2 Molecular electrostatic potential

MEPs have proved useful in determining the relative polarity of compounds as well as providing essential information on molecular charge distribution patterns. As a result, studying the MEP of the examined compounds may provide insight into their electrophilic and nucleophilic cores (Figure 2). It is worth mentioning that molecular electrostatic potential data can be classified using traditional color codes. The electron-rich centers are indicated by a red color scheme, which symbolizes the highest negative electrostatic potential. On the other hand, a blue hue region denotes electron-deficient areas (i.e., the most positive electrostatic potential). The light blue, yellow, and green moieties represent a molecule’s region of slightly electron-deficient cores, marginally electron-rich areas, and zero electrostatic potential potions, respectively. We can deduce that a molecule’s potential declines in the following order based on the color scheme: blue > light blue > green > yellow > red. Figure 2 represents molecular electrostatic potential maps of lonchocarpol A, broussonol E, diplacol, and dexamethasone. There is a clear maximum concentration of electrons located at the alkyl groups and oxygen atoms of lonchocarpol A attached to the diphenyl groups.

In contrast, the region of most positive electron potential of lonchocarpol A is located at the hydrogen atoms of the methyl group of the phenyl group. The most negative potential for dexamethasone is situated on the imidazole rings’ two-hydroxyl group and oxygen atoms. Broussonol E recorded the highest negative electrostatic potential, including multiple hydroxyl group points. All the compounds have been reported for their biological and chemical properties. Therefore, MEP provides detailed insights into the molecular charge distribution clusters in the studied compound.

### 3.3 Mulliken population analysis

Table 2 shows the atomic charge distribution of lonchocarpol A, broussonol E, diplacol, and dexamethasone computed *via* the Mulliken population analysis using the PM7-based semi-empirical Hamiltonian calculations.

**TABLE 2:** Calculated Mulliken atomic charges of the top four compounds.

Atom No	Atom (lonchocarpol A)	Mulliken charge (lonchocarpol A)	Atom (broussonol E)	Mulliken charge (broussonol E)	Atom (diplacol)	Mulliken charge (diplacol)	Atom (dexamethasone)	Mulliken charge (dexamethasone)
1	O	−0.44443	O	−0.34135	O	−0.43335	F	−0.22161
2	O	−0.52506	O	−0.5126	O	−0.57712	O	−0.57211
3	O	−0.49887	O	−0.48847	O	−0.5101	O	−0.59853
4	O	−0.59159	O	−0.45938	O	−0.57763	O	−0.47831
5	O	−0.51036	O	−0.59099	O	−0.49375	O	−0.5496
6	C	0.207278	O	−0.53436	O	−0.47351	O	−0.50928
7	C	**−0.64918** ^a^	O	−0.48846	O	−0.47101	C	0.119523
8	C	0.553263	C	**−0.68688** ^a^	C	0.111928	C	−0.17951
9	C	**−0.73661** ^a^	C	−0.45146	C	−0.09313	C	−0.19425
10	C	−0.37697	C	0.553329	C	**−0.73622** ^a^	C	0.167313
11	C	**0.702303** ^b^	C	**0.602767** ^b^	C	**0.601256** ^b^	C	0.126795
12	C	−0.45972	C	0.219836	C	0.597236	C	−0.40441
13	C	0.597616	C	0.012863	C	−0.03862	C	−0.51737
14	C	0.506552	C	−0.21543	C	−0.4738	C	−0.07233
15	C	−0.20439	C	0.532411	C	**0.611783** ^b^	C	0.110405
16	C	−0.21769	C	**0.620079** ^b^	C	**−0.6357** ^a^	C	−0.00231
17	C	−0.2394	C	−0.02945	C	−0.23452	C	−0.32459
18	C	−0.06819	C	**−0.60901** ^a^	C	0.561417	C	**−0.62434** ^a^
19	C	−0.01567	C	−0.06649	C	−0.33019	C	−0.41779
20	C	−0.32883	C	−0.22206	C	−0.14207	C	0.177623
21	C	−0.35013	C	−0.3368	C	−0.36668	C	0.407181
22	C	−0.35526	C	−0.25838	C	0.123515	C	**−0.60478** ^a^
23	C	−0.44732	C	0.104187	C	−0.39687	C	**−0.60459** ^a^
24	C	0.158178	C	0.234676	C	0.215686	C	−0.11383
25	C	0.371538	C	−0.37461	C	−0.34313	C	−0.52143
26	C	0.137273	C	−0.34269	C	−0.29078	C	−0.23229
27	C	**−0.64618** ^a^	C	0.210516	C	0.227416	C	−0.43236
28	C	**−0.65128** ^a^	C	0.149934	C	−0.65057	C	**0.621716** ^b^
29	C	**−0.64474** ^a^	C	−0.66075	C	−0.33623	H	0.174307
30	C	**−0.65366** ^a^	C	−0.66589	C	0.161681	H	0.199263
31	H	0.177918	C	−0.64924	C	−0.65057	H	0.18274
32	H	0.260334	C	−0.65697	C	−0.64239	H	0.202663
33	H	0.257829	H	0.202691	H	0.209879	H	0.217255
34	H	0.180081	H	0.147876	H	0.229331	H	0.223055
35	H	0.186761	H	0.290018	H	0.291301	H	0.155531
36	H	0.170022	H	0.224598	H	0.172566	H	0.197823
37	H	0.214067	H	0.192595	H	0.212728	H	0.181412
38	H	0.193514	H	0.204748	H	0.248627	H	0.185519
39	H	0.206486	H	0.264391	H	0.222298	H	0.21967
40	H	0.201087	H	0.195052	H	0.216964	H	0.193173
41	H	0.217387	H	0.21727	H	0.371795	H	0.211806
42	H	0.413892	H	0.41358	H	0.196626	H	0.200817
43	H	0.376606	H	0.367127	H	0.185069	H	0.208468
44	H	0.242263	H	0.37573	H	0.218915	H	0.191525
45	H	0.226644	H	0.214546	H	0.170655	H	0.211997
46	H	0.204763	H	0.210442	H	0.176395	H	0.217508
47	H	0.204729	H	0.206894	H	0.41009	H	0.209823
48	H	0.202827	H	0.205874	H	0.220548	H	0.221053
49	H	0.229196	H	0.236009	H	0.200566	H	0.211325
50	H	0.203416	H	0.209537	H	0.214513	H	0.362716
51	H	0.199179	H	0.378383	H	0.382286	H	0.222359
52	H	0.207816	H	0.210921	H	0.194678	H	0.354091
53	H	0.210879	H	0.213816	H	0.349727	H	0.252387
54	H	0.209347	H	0.21176	H	0.347314	H	0.231837
55	H	0.208034	H	0.206996	H	0.207703	H	0.204019
56	H	0.20602	H	0.219071	H	0.210102	H	0.244501
57	H	0.216366	H	0.211674	H	0.204851	H	0.356402
58	H	0.354056	H	0.36951	H	0.207125	—	—
59	—	—	—	—	H	0.205716	—	—
60	—	—	—	—	H	0.207643	—	—

a Most negatively charged region.

b Most positively charged region.

Because atomic charges affect compounds’ molecular and electrical characteristics, estimating each compound’s partial atomic charges is critical for understanding the charge distribution. Calculating the atomic charges of any small molecule ligand can be used to calculate the adsorptive centers. Table 2 shows that the examined structures’ oxygen and carbon atoms have electron-rich chemical species (i.e., they have the most negative electronic charges), which may be due to their molecular relaxation. However, the predominant positive charge regions were observed to be covered by carbon atoms, despite the fact that some carbon atoms in the investigated compounds possessed negative atomic charges. Table 2 shows that the atoms O7, C9, and C27–30 of lonchocarpol have the most negative atomic charges, whereas the C11 and C16 of broussonol E have the highest negative atomic charges in lonchocarpol A. In terms of broussonol E, C10 and C16 were observed for negatively charged atoms, whereas C11 and C15 occupied positive regions. Dexamethasone’s ionic structure established negatively charged electrostatic contacts with C18 and C22–23 while demonstrating C27 as the only positive electrostatic atoms. Overall, it can be deduced that there are variations between the atoms of the studied compounds occupying positive and negative regions. This is also supported by the difference between the compound’s inhibitory potential and their chemical stability.

### 3.4 Non-linear optics analysis

NLO materials have played an important role in contemporary technologies, providing various industrial and medicinal benefits, some of which have been detailed in prior studies (Muhammad et al., 2021). The most prominent quality of analyzing NLO properties from a fascinating perspective on chemical methodologies and applications is their tendency to provide considerable insights into how small changes in molecular structures might alter NLO responses. Tables 3, 4 present and summarize various NLO responses and their components for lonchocarpol A, broussonol E, diplacol, and dexamethasone estimated using the PM7 semi-empirical Hamiltonian calculations in MOPAC 2016. The dipole moment (*µ*) gives information on a bond’s or molecule’s ionic character state. Ionic property is generally associated with molecules with a higher dipole moment value. Furthermore, dipole moments are crucial in forecasting a molecule’s structure and reactivity. The dipole moments for the studied compounds, lonchocarpol A, broussonol E, diplacol, and dexamethasone, were 2.663, 4.122, 5.209, and 5.334, respectively. The computation of polarizability (α_0_) and hyperpolarizability (β_0_ and γ_0_) in molecular systems are helpful in describing charge delocalization and measuring NLO effects (Maragatham et al., 2019). More intriguingly, they have been used in pharmaceutical development. The coefficients in the Taylor series expansion depending on the energy in the external electric field (Muthu et al., 2014; Maragatham et al., 2019) are denoted as the first hyperpolarizability (β_0_) and associated properties (µ, α_0_, and γ_0_) of the described compounds: lonchocarpol A, broussonol E, diplacol, and dexamethasone. The expansion can be expressed as follows for a weak homogenous external electric field:

E=E0−∑−^1/2∑−j^^1/6∑−jk^^^1/24∑−jkl^^^^.... . (13)

**TABLE 3:** Non-linear optics (NLO) measurements of the top four compounds.

Parameters	Lonchocarpol	Broussonol E	Diplacol	Dexamethasone (control)
Dipole moment (Debye)				
*µ*_x_	−0.016	0.936	5.075	1.003
*µ*_y_	2.546	4.004	1.089	1.943
*µ*_z_	0.781	−0.284	0.444	4.865
*µ*	2.663	4.122	5.209	5.334
Polarizability (a.u.)				
*α*_xx_	423.2262	422.1966	474.0150	276.5705
*α*_xy_	−32.1733	422.1966	20.7672	−1.9526
*α*_yy_	321.3188	415.5835	337.6663	249.1648
*α*_xz_	5.8622	5.8562	11.7997	−38.5436
*α*_yz_	12.2187	38.3700	32.3409	6.6118
*α*_zz_	252.3939	230.5271	228.9737	308.3070
*α*_0_	332.31294	356.10239	346.88497	278.01414
Hyperpolarizability (a.u.)				
*ß*_xxx_	−828.11534	−1,758.35361	−857.13155	−105.90769
*ß*_xxy_	1,274.91556	1,770.96251	1,087.95116	−11.22939
*ß*_xyy_	408.19522	−939.40438	392.33997	−56.47036
*ß*_yyy_	−205.66502	198.04721	−145.10306	−0.22422
*ß*_xxz_	218.15245	622.87825	−23.37222	−19.56624
*ß*_xyz_	−56.15189	19.08646	31.58709	14.60703
*ß*_yyz_	−40.72098	−90.27506	22.36855	30.11794
*ß*_xzz_	67.05907	86.15574	8.42037	164.77391
*ß*_yzz_	−12.20923	−74.49353	9.07706	−12.27861
*β*_0_	678.2379	1946.6357	633.4487	83.4142
*γ*_xxxx_	136,082.88516	437,948.46962	122,946.68736	13,932.57214
*γ*_yyyy_	40,946.55150	86,024.86024	18,258.69096	14,983.28635
*γ*_zzzz_	6,622.48729	6,562.67994	3,978.16272	15,251.43212
*γ*_xxyy_	49,373.55773	208,421.66382	48,042.22109	4,810.78759
*γ*_xxzz_	8,548.39107	18,233.01061	11,992.55351	4,799.66326
*γ*_yyzz_	4,755.42360	11,210.83548	2,029.91003	7,556.43105
*γ*_0_	61,448.83940	205,675.48909	53,862.59096	15,788.15525

Standard value for urea (µ = 1.3732 Debye; *ß*_0_ = 0.3728 × 10^−30^ esu); esu, electrostatic unit. (For α, 1 a.u is equal to 0.1482 × 10^–24^ esu. Similarly, for β, 1 a.u is equal to 8.6393 × 10^−33^ esu).

**TABLE 4:** Molecular electric dipole moment (*µ*), static polarizability (*α*_0_), static first-order hyperpolarizability (*ß*_0_), and static second-order hyperpolarizability (*γ*_0_) of the top four compounds.

Compound	Dipole moment (Debye)	Static polarizability (α_0_ × 10^−23^ esu)	Static first-order hyperpolarizability (*ß*_0_ × 10^−30^ esu)	Static second-order hyperpolarizability (γ_0_ × 10^−39^ esu)
Lonchocarpol A	2.663	5.1226	0.0586	30,949.9528
Broussonol E	4.122	5.2774	0.0017	103,592.6267
Diplacol	5.209	5.1654	0.0590	32,680.4170
Dexamethasone (control)	5.334	4.1202	0.7207	7,952.0242

Note that E_0_ describes the energy of the unperturbed molecules; Fi represents the field at the origin; and µ_i_, α_ij_, β_ijk_, and γ_ijkl_ correlate to the dipole moment, static polarizability, first-order hyperpolarizability, and second order hyperpolarizability, respectively. The total dipole moment µ, static mean polarizability α_0_, mean first-order hyperpolarizability β_0_, and second order hyperpolarizability γ_0_ can be estimated by the following equations:

$$\text{Dipole moment }\mu =\sqrt{\mu_{x}^{2}+\mu_{y}^{2}+\mu_{z}^{2}};$$ (14)

$$\text{Static mean polarizability }\alpha_{\text{0}}=\left(\alpha_{xx}+\alpha_{yy}+\alpha_{zz}\right)/3;$$ (15)

$$\text{Static first order hyperpolarizability β}=\sqrt{\beta_{x}^{2}+\beta_{y}^{2}+\beta_{z}^{2}};$$ (16)

$$\text{where }\beta_{\text{x}}=\frac{3}{5}\left(\beta_{xxx}+\beta_{xyy}+\beta_{xzz}\right),$$ (17)

$$\beta_{\text{y}}=\frac{3}{5}\left(\beta_{yyy}+\beta_{yzz}+\beta_{yxx}\right),$$ (18)

$$\beta_{\text{z}}=\frac{\;3}{5}\left(\beta_{zzz}+\beta_{zxx}+\beta_{xyy}\right),$$ (19)

$$\beta_{\text{Total}}=\sqrt{{\left(\beta_{xxx}+\beta_{xyy}+\beta_{xzz}\right)}^{2}+{\left(\beta_{yyy}+\beta_{yzz}+\beta_{yxx}\right)}^{2}+{\left(\beta_{zzz}+\beta_{zxx}+\beta_{xyy}\right)}^{2}},$$ (20)

$$\mathrm{\gamma =}\frac{1}{5}\left[\gamma_{xxxx}\gamma_{yyyy}\gamma_{zzzz}+2\left(\gamma_{xxxx}+\gamma_{yyyy}+\;\gamma_{zzzz}\right)\right].$$ (21)

Notably, any compound with a higher value of first-order hyperpolarizability denotes an NLO active compound and vice versa. Table 4 shows that the hyperpolarizability value of dexamethasone is (0.7207 × 10–30), which is 10 times higher than that of lonchocarpol A (0.0586 × 10–30), broussonol E (0.0017 × 10–30), and diplacol (0.0590 × 10–30). Collectively, this study proposed that dexamethasone is the most suitable compound for NLO-based technology.

### 3.5 Molecular docking and binding site analysis

#### 3.5.1 Inhibitory potential of promising phytodrugs against SARS-CoV-2 spike glycoprotein, 3CLpro, PLpro, and RdRp

The 3CLpro, also referred to as NSP5, mediates the maturation of Nsps, which is vital in the virus’s lifecycle. The structural analysis and catalytic mechanism of 3Clpro using biophysical techniques have been widely investigated (Pillaiyar et al., 2016). Therefore, 3CLpro remained an important therapeutic target for developing potential anti-coronavirus drug candidates. Peptide inhibitors and small molecules are inhibitors targeting the SARS-CoV-2 3CLpro. From the molecular docking result, various molecular interactions, including hydrogen bonding, hydrophobic, polar, and pi–pi interactions, were observed and analyzed while ranking the compounds based on their binding poses. Although, nicotiflorin, schaftoside, acetoside, and mallophenol demonstrated average binding energy of −11.20 kcal/mol (Table 5). They were eliminated from further studies because of their undruggable properties. Interestingly, lonchocarpol A, broussonol E, diplacol, and dexamethasone (reference compound) were selected for further analysis because of drug-like properties, molecular interactions, and high binding energy.

**TABLE 5:** Binding energy of compounds library against the SARS-CoV-2 therapeutic target.

S/NS	Compounds	Spike glycoprotein RBD (6MOJ) (kcal/mol)	Compounds	3CLpro (6M2N) (kcal/mol)	Compounds	PLpro (7CJM) (kcal/mol)	Compounds	RdRp (7D4F) (kcal/mol)
1	Rutin	−10.941	Nicotiflorin	−11.442	Aucubin	−8.767	Acteosides	−10.632
2	Delphinidin 3-O-beta-D-sambubioside	−10.709	Schaftoside	−11.389	Rutin	−8.698	Cynaroside	−9.193
3	Hesperidin	−10.627	Acteoside	−11.291	Nicotiflorin	−8.685	Hydroxycitric acid	−9.087
4	Acteoside	−10.033	Mallophenol B	−11.226	Mallophenol B	−8.106	Rutin	−8.704
5	Kuromanin	−9.902	Kolaflavanone	−10.496	Hesperidin	−7.671	Schaftoside	−8.347
6	Pelargonidin 3-glucoside	−9.684	Aucubin	−10.295	Cynaroside	−7.537	Bergenin	−8.127
7	Lauroside E	−9.599	Tanariflavanone C	−10.278	Kuromanin	−7.186	Kuromanin	−7.687
8	Nicotiflorin	−9.447	(+)-Gallocatechin gallate	−10.334	Schaftoside	−7.114	Mallophenol B	−7.680
9	Diplacol	−8.733	Delphinidin 3-O-beta-D-sambubioside	−10.035	Pelargonidin 3-glucoside	−6.811	Lauroside E	−7.641
10	Myricetin	−8.725	Rutin	−8.987	Nymphaeol B	−6.687	Hydroxycitric acid	−7.629
11	Nymphaeol B	−8.291	Luteolin	−8.866	(+)-Gallocatechin gallate	−6.663	Pelargonidin 3-glucoside	−7.489
12	Schaftoside	−8.251	Nymphaeol C	−8.748	Macaranone A	−6.548	(+)-Gallocatechin gallate	−8.937
13	(+)-Gallocatechin gallate	−8.289	Macakurzin A	−8.723	Myricetin	−6.531	Delphinidin 3-O-beta-D-sambubioside	−7.333
14	Macakurzin A	−8.191	Isovitexin	−8.710	Bergenin	−6.378	Gallic acid	−7.122
15	Tanariflavanone D	−8.160	**Lonchocarpol A** ^a^	**−8.644** ^a^	Quercetin	−6.377	Lonchocarpol A	−7.017
16	Chlorogenic acid	−8.131	**Diplacol** ^a^	**−8.576** ^a^	Isovitexin	−6.277	Nicotiflorin	−6.819
17	Isovitexin	−8.096	Tomentosanol D	−8.470	Lonchocarpol A	−6.161	Aucubin	−6.752
18	Cynaroside	−7.991	Isolicoflavonol	−8.451	Lauroside E	−6.117	Tanariflavanone D	−6.739
19	Quercetin	−7.996	Fisetin	−8.459	Acteoside	−6.115	Broussonol E	−6.342
20	Bonnaniol	−7.904	Denticulaflavonol	−8.253	Nymphaeol A	−6.047	Macarangioside F	−6.255
21	Macakurzin A	−7.884	Glepidotin A	−8.231	Diplacol	−5.972	Chlorogenic acid	−6.089
22	Aucubin	−7.818	Myricetin	−8.187	Catalpol	−5.733	Protocatehuic acid	−6.019
23	Broussonol E	−7.490	Catalpol	−8.164	Tomentosanol D	−5.692	Diplacol	−5.997
24	Mallophenol B	−7.425	Macarangin	−8.134	Broussonol E	−5.674	Cianidanol	−5.992
25	Luteolin	−7.414	Cynaroside	−8.066	Macakurzin A	−5.631	Tomentosanol D	−5.875
26	Lonchocarpol A	−7.331	**Broussonol E** ^a^	**−8.069** ^a^	Isolicoflavonol	−5.602	Isovitexin	−5.871
**27**	Dexamethasone	−5.641	**Dexamethasone** ^a^	**−5.302** ^a^	Dexamethasone	−3.939	Dexamethasone	−2.946

a Selected compounds and their binding energy against 3CLpro for further molecular dynamics analysis.

Lonchocarpol A is a flavone obtained from *Lonchocarpus* and *Erythrina* species and has been reported for its biological activities, including anticancer, insecticidal, and antibacterial activity, amongst others. Interestingly, lonchocarpol A has also been synthesized using various synthetic methods and have also received significant interest as a compound with numerous therapeutic benefits (Salvatore et al., 1998). Lonchocarpol A has a binding affinity of −8.644 kcal/mol and hydrogen bond interactions with ARG188 based on its side hydroxyl group. All significant interactions exhibited by the compound were mainly due to its alkyl side group and phenyl ring.

**FIGURE 3:**
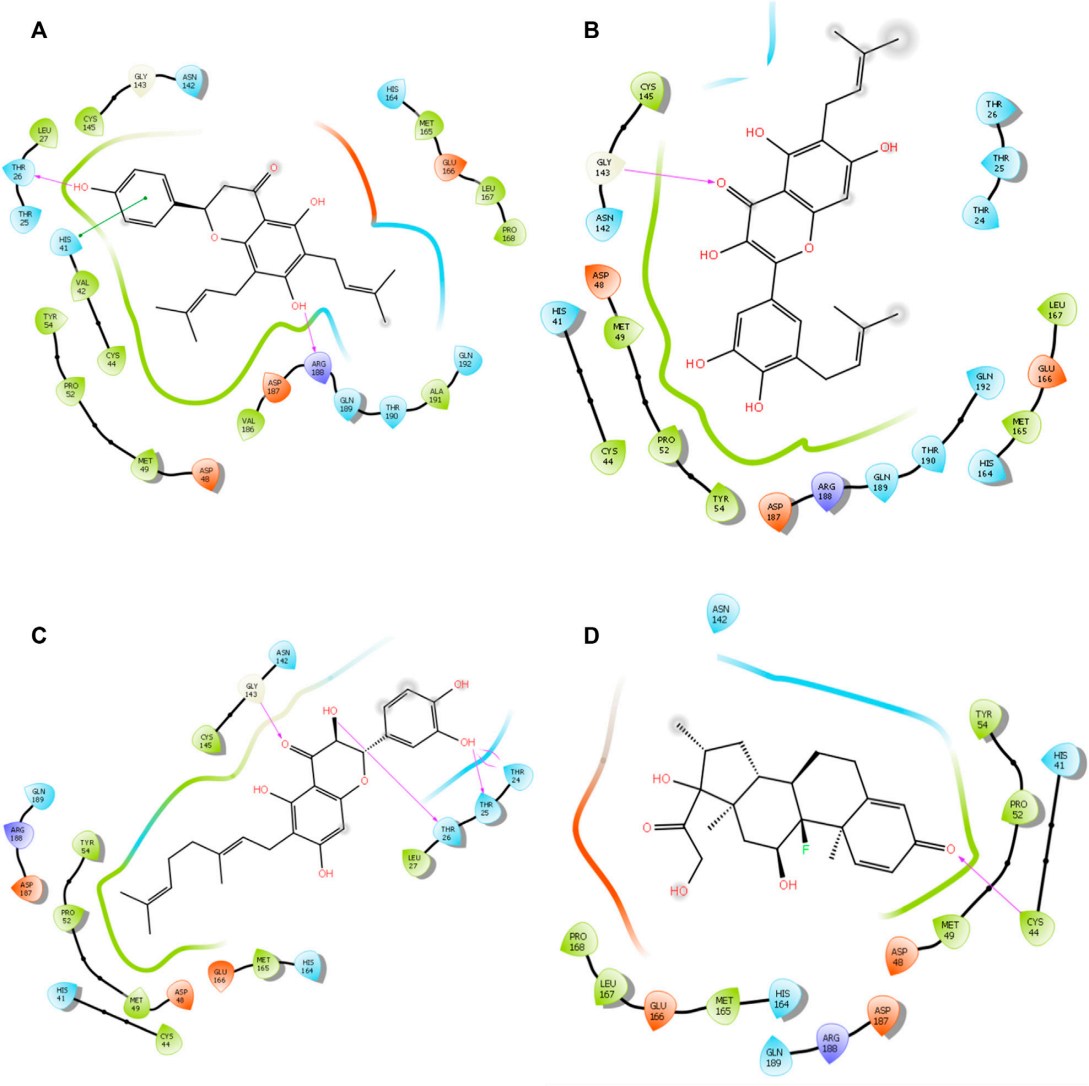
3D interaction of top four docked complexes: **(A)** lonchocarpol **A–**3CLpro complex, **(B)** broussonol **E**–3CLpro complex, **(C)** diplacol–3CLPro, **(D)** dexamethasone–3CLPro.

The alkyl groups present in the phenyl moiety interact with hydrophobic amino acids TYR54, PRO52, MET49, CYS44, VAL42, LEU27, CYS145, VAL186, ALA191, LEU167, PRO168, and MET165, and polar amino acids HIS41, ASN142, GLN189, THR190, GLN192, and HIS164 (Table 6). The other notable interactions were pi–pi/charge interactions between the aromatic ring of lochocarpol A with ASP48, ASP187, GLU166, and ARG188 (Figure 3). The second selected lead compound, diplacol, showed a similar hydrogen bond with amino acid ARG188 as in lonchocarpol A; however, the two dihydroxylphenyl and the alky group of the compounds were responsible for its hydrophobic interactions with various amino acid residues (TYR54, PRO52, CYS44, MET49, MET165, CYS145, and LEU27) at the 3CLpro active site. The polar protein–ligand interactions exhibited by diplacol follow the same pattern as lonchocarpol A with subtle differences. Diplacol established polar interactions with the following amino acid residues: GLN189, HIS41, HIS164, ASN142, THR26, THR25, and THR24; these residues were present at the binding pocket of 3CLpro. Broussuonol E has a binding energy of −8.069 kcal/mol and shows key biomolecular interactions with certain amino acids such as THR26, THR25, THR24, HIS164, GLN192, THR190, GLN189, HIS41, and ASN142 within the 3CLpro active site. Broussuonol E demonstrated other interactions, such as polar and pi–pi interactions. The reference compound (dexamethasone) has the least binding energy with similar inter- and intramolecular interactions with the lead compounds. Notably, dexamethasone interacted with PRO52, TYR54, MET165, MET49, and CYS44 amino acid residues, which were also recorded in the lead compounds interactions. Therefore, the top three compounds were proposed to have a similar mechanism of action as dexamethasone since they share the same amino acid interactions with the targets.

**TABLE 6:** Molecular interaction profiling and docking score of top four compounds.

S/N	II interacting amino acid residues							
	Lead compounds against 3CLPro	Docking score (kcal/mol)	H-bond	Hydrophobic	Polar	Charged (negative)	Charged (positive)	Glycine
1	Lonchocarpol A	−8.644	ARG188	TYR54, PRO52, MET49, CYS44, VAL42, LEU27, CYS145, VAL186, ALA191, LEU167, PRO168, MET165	HIS41, ASN142, GLN189, THR190, GLN192, HIS164	ASP48, ASP187, GLU166	ARG188	GLY143
2	Diplacol	−8.576	ARG188	TYR54, PRO52, CYS44, MET49, MET165, CYS145, LEU27	GLN189, HIS41, HIS164, ASN142, THR26, THR25, THR24	ASP48, GLU166, ASP187	ARG188	GLY143
3	Broussonol E	−8.069	ARG188	CYS145, CYS44, MET49, PRO52, TYR54, MET165, LEU167	THR26, THR25, THR24, HIS164, GLN192, THR190, GLN189, HIS41, ASN142	ASP48, ASP187, GLU166	ARG188	GLY143
4	Dexamethasone	−5.302	ARG188	PRO168, LEU167, MET165, MET49, CYS44, PRO52, TYR54	HIS164, GLN189, HIS41, ASN142	GLU166, ASP48, ASP187	ARG188	—

Spike is coronavirus’s major structural protein, which assembles as a trimer into a unique corolla structure on the virus’s surface. The spike protein mediates the virus interaction with the host cell by binding to the host angiotensin-converting enzyme (ACE-2). Certain host cell proteases, such as TMPRSS2, cleave the spike protein into two subunits, S1 and S2, which play a crucial role in receptor recognition and cell membrane fusion (Millet and Whittaker, 2015). Therefore, blocking the coronavirus entry into the cell by targeting the spike glycoprotein has significantly been harnessed in the development of therapeutic agents against coronavirus. From the virtual screening results, rutin, delphinidin 3-O-beta-D-sambubioside, and hesperidin show the highest binding energy of −10.941, −10.709, and −10.627 kcal/mol, respectively. Unfortunately, these compounds failed the toxicity assessment and were eliminated from a further study. However, the leads compounds, namely, diplacol E, broussonol E, and lonchocarpol A, have a binding affinity of −8.733, −7.490, and −7.331 kcal/mol against spike glycoprotein, respectively. The protein–ligand contacts show that the lead compounds established some essential hydrogen and hydrophobic interactions. It can be deduced that lonchocarpol has the lowest binding energy against spike glycoprotein when compared to the remaining two lead compounds (diplacol and broussonol E).

Papain-like proteinase (Plpro) plays a role in the cleavage of the N-terminus of the replicase polyprotein to produce non-structural proteins, including Nsp1, Nsp2, and Nsp3, which are involved in the virus replication (Li et al., 2016). Thus, based on the vital role played by PLpro in virus replication and infection, it has received intense consideration as a therapeutic target for coronavirus inhibitors. There have been no FDA-approved inhibitors of PLpro. Aucubin was recorded with the highest binding energy against PLpro, with −8.767 kcal/mol. Aucubin’s high binding energy may be attributed to its structural basis, including its imidazole ring. Nicotiflorin optimally occupied the binding pocket of the target (PLpro), which may be attributed to its ring system. The presence of multiple hydroxyl groups at the nicotiflorin structures establishes intermolecular hydrogen bonds. Several docked compounds, including rutin, diplacol, hesperidin, and kuromanin, showed high binding energy against PLpro while demonstrating pi–pi and hydrophobic interactions with amino acid residues at the active site of PLpro. The lead compounds lonchocarpol A, diplacol, and broussonol E have a binding energy of −6.161, −5.972, and −5.674 kcal/mol, respectively, when docked into the binding pocket of PLpro. The binding energy against PLpro follows a similar pattern to that of 3CLpro. However, the lead compounds’ structural poses, binding energy, and molecular interactions against PLpro are relatively low when compared to that 3CLpro.

RNA-dependent RNA polymerase (RdRp: NSP 12) is a conserved protein in coronavirus with a primary function in the coronavirus replication/transcription complex. Targeting NSp-12RdRp has been well documented for its little to no side effects on the host cell (Ruan et al., 2020). However, there has been no specific RdRp inhibitor till present. The crystal structure of RdRp was downloaded and refined for the protein–ligand docking process. Molecular docking results of RdRp following the extra-precision approach show the antiviral potential of the docked compounds.

Interestingly, acetosides and cynarosides demonstrated the highest binding energy of −10.632 and −9.193 kcal/mol, respectively. However, they were not selected for further analysis because of their rule of five violations. The lead compounds (lonchocarpol A, diplacol, and broussonol E) and the reference compound (dexamethasone) had a very low binding energy against the RdRp target; this may be attributed to the configuration and nature of the RdRp active site and its amino acid residues. The molecular docking results predicted that the lead compounds could stop the viral replication of SARS-CoV-2 through their inhibitory potential. Lonchocarpol A exhibited a docking score of −7.017 kcal/mol when docked into the active site of RdRp. Broussonol E has a binding energy of −6.342 kcal/mol, followed by diplacol with a binding energy of −5.997 kcal/mol. Although, other screened small molecules such as kuromanin, lauroside E, gallic acid, chlorogenic acid, and isovitexin demonstrated relatively high binding energy of −7.687, −7.641, −7.122, −6.089, and −5.871 kcal/mol, respectively.

### 3.6 MM-GBSA binding energy of top inhibitors

**FIGURE 4:**
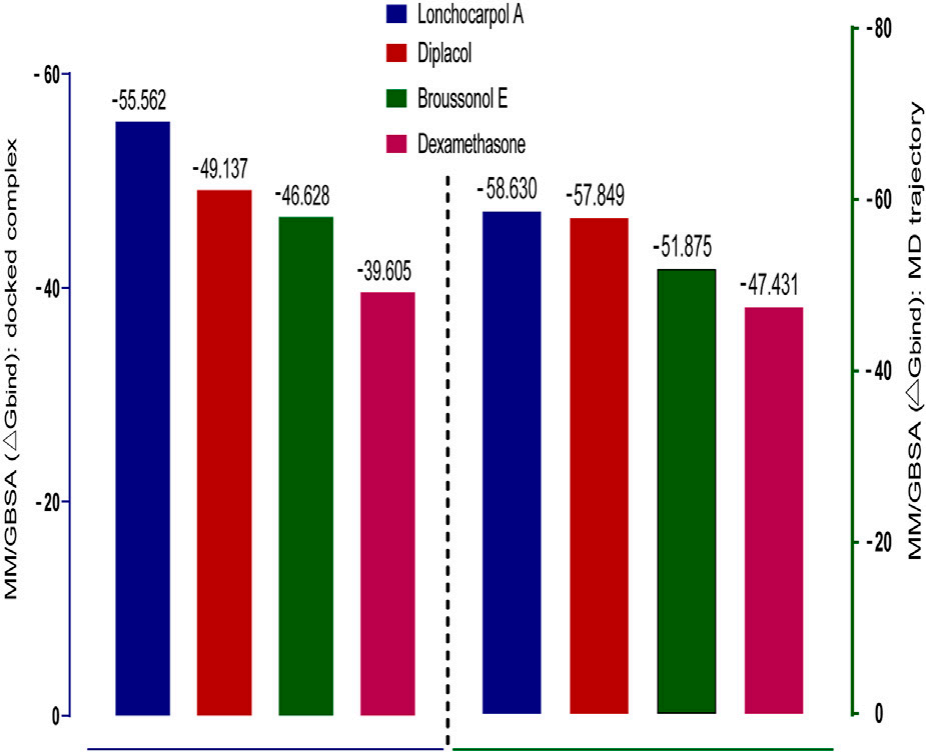
Graphical representation of Prime/MM-GBSA binding energy (∆G_bind_) for docked complex and MD trajectory. The left frame (blue) denotes the post-docking MM-GBSA binding energy, whereas the right frame (green) signifies the MM-GBSA binding energy of post-simulation analysis.

Molecular mechanics generalized Born surface area (MM-GBSA) has been widely explored as an advanced computational approach to analyze binding energy with an improved algorithm and solvation model. Compared to docking, post-scoring compounds using MM-GBSA have been demonstrated to correlate better to their reported binding affinity of docked complexes (Greenidge et al., 2013; Tripathi et al., 2013). The MM-GBSA method is more accurate in estimating the free binding energies of protein–ligand complexes than docking scores. A post-docking MM/GBSA analysis of the docked complexes was −55.562, −49.137, −46.628, and −39.605 kcal/mol for lonchocarpol A, broussonol E, diplacol, and dexamethasone, respectively, as shown in Figure 4. The post-simulation MM/GBSA, which further validates the binding affinity of the compounds, shows similar binding energy to the post-docking analysis.

### 3.7 Drug likeness and toxicity descriptors prediction

Pharmacokinetic properties of the top four lead potential antiviral flavonoids were predicted, studied, and tabulated, as shown in Table 7. It is clear that except (-) lonchocarpol A and broussonol E, none penetrated the blood–brain barrier. Under the adsorption and distribution, the Caco-2 permeability of the lead compounds shows that lonchocarpol A and dexamethasone showed positive ions of Caco-2^−^ permeability whereas diplacol and broussonol E showed negative ion of Caco-2^−^ permeability.

**TABLE 7:** Pharmacokinetics profile of top four compounds.

Models	Lonchocarpol A	Diplacol	Broussonol E	Dexamethasone
Absorption and distribution				
Blood–brain barrier	BBB−	BBB+	BBB−	BBB+
Caco-2 permeability	Caco-2^+^	Caco-2^−^	Caco-2^−^	Caco-2^+^
P-glycoprotein (substrate)	Non-substrate	Non-substrate	Non-substrate	Substrate
P-glycoprotein (inhibitor)	Inhibitor	Inhibitor	Inhibitor	Non-inhibitor
LogS (aqueous solubility)	−3.925	−4.285	−3.567	−3.703
Renal organic cation transporter 2 (OCT2)	Non-inhibitor	Non-inhibitor	Non-inhibitor	Inhibitor
Metabolism				
CYP450 2C9 (substrate)	Non-substrate	Non-substrate	Non-substrate	Non-substrate
CYP450 2C9 (inhibition)	Inhibitor	Inhibitor	Inhibitor	Non-inhibitor
CYP450 2D6 (substrate)	Non-substrate	Non-substrate	Non-substrate	Non-substrate
CYP450 2D6 (inhibition)	Non-inhibitor	Non-inhibitor	Non-inhibitor	Non-inhibitor
CYP450 3A4 (substrate)	Substrate	Non-substrate	Non-substrate	Substrate
CYP450 3A4 (inhibition)	Non-inhibitor	Inhibitor	Inhibitor	Non-inhibitor
CYP450 1A2	Inhibitor	Inhibitor	Inhibitor	Non-inhibitor
CYP450 2C19	Inhibitor	Inhibitor	Inhibitor	Non-inhibitor
Toxicity				
Ames toxicity	Non-toxic	Toxic	Non-toxic	Non-toxic
hERG inhibition	Inhibitor	Inhibitor	Inhibitor	Inhibitor
Carcinogenicity	Non-carcinogenic	Non-carcinogenic	Non-carcinogenic	Non-carcinogenic
Acute oral toxicity	III	III	III	III
Rat LD50	2.705	2.591	2.129	2.189

The action of the four lead compounds on the P-glycoprotein (substrate) showed that lonchocarpol A, diplacol, and broussonol E are non-substrate. In contrast, only dexamethasone showed the level of a substrate to the glycoprotein. Lonchocarpol A, diplacol, and broussonol E are promising glycoprotein inhibitors from COVID-19, whereas only dexamethasone shows its non-inhibiting property. About the LogS (aqueous solubility), broussonol E has the least solubility with −3.567, followed by dexamethasone with −3.703, which is greater than broussonol E; lonchocarpol A has a solubility value in the aqueous range of −3.925, and the highest solubility value out of the four lead compounds in the aqueous state is diplacol with a value of −4.285. All the compound complexes exhibit non-inhibitor on renal organic cation transporter 2 (OCT2), except dexamethasone, which shows inhibiting properties.

For the metabolism, the CYP450 2C9 (substrate) and CYP450 2D6 (substrate) showed that all the four lead compounds are non-substrate in nature, and CYP450 2D6 (inhibition) showed the lead compounds as non-inhibitors. CYP450 2C9 (inhibition), CYP450 1A2, and CYP450 2C19 showed that three lead compounds are natural inhibitors, whereas only dexamethasone was non-inhibitor in nature.

For the Ames toxicity, all three lead compounds are non-toxic, whereas only the diplacol is toxic. In the analysis of hERG inhibition and carcinogenicity, all lead compounds exhibit inhibiting properties and non-carcinogenic ability. The Rat LD50 is higher on lonchocarpol A with a value point of 2.705 and lower on broussonol with a value of 2.129. Thus, natural phytocompounds are not naturally occurring and reported negligible toxicity when tested *in vitro*; hence, it could be a promising drug candidate and can be tested *in vitro* then *in vivo*. Lethal doses (LD50) of all the natural compounds were higher when compared to chemical drugs, which denotes that even at a higher dosage natural compounds are less toxic than chemically synthesized drugs. Thus, chemical drugs are toxic from the pharmacokinetic predictions compared to natural compounds; natural compounds have shown potential against several diseases with the least side effects (Benfenati et al., 2009). The drug-likeness properties (Table 7) of the compounds show they are druggable compounds with no violations of Lipinski’s assessment and Verber’s rules (Table 8). Some classes of compounds have been reported for their false-positive results during virtual screening. These compounds are referred to as Pan-assay interference compounds (PAINS). Chemical compounds in this category have been found to target numerous biological targets rather than a specific target (Baell, 2016). Catechols, quinones, curcumin, and toxoflavin are common examples of PAINS (Baell and Walters, 2014). PAINS screening of our compounds was carried out using the SwissADME web server (Daina et al., 2017), and the results shows that diplacol and broussonol E are PAINS compounds because of their substructural motifs with catechols. However, lochocarpol A and the reference compound (dexamethasone) will produce specific molecular interactions because they do not belong to the PAINS class.

**TABLE 8:** Drug-likeness prediction of top four compounds.

Compounds	MW	HBA	HBD	Veber’s rule	Violation of ROF	Pains
Lonchocarpol A	408.49 g/mol	5	3	TPSA = 86.99 Å^2^ Num. rotatable bonds = 5	0	0 alert
DIplacol	440.49 g/mol	7	5	TPSA = 127.45 Å^2^ Num. rotatable bonds = 6	0	1 alert (catechol)
Broussonol E	438.47 g/mol	7	5	TPSA = 131.36 Å^2^ Num. rotatable bonds = 5	0	1 alert (catechol)
Dexamethasone (reference compound)	392.46 g/mol	6	3	TPSA = 94.83 Å^2^ Num. rotatable bonds = 2	0	0 alert

MW, molecular weight; HBA, hydrogen bond acceptor; HBD, hydrogen bond donor; TPSA, topological surface area; ROF, rule of five; PAINS, pan-assay interference structure.

### 3.8 Molecular dynamics simulation of the complexes

Molecular dynamics (MD) simulation is an essential tool that helps in the study of macromolecules such as nucleosomes, ribosomes, membrane proteins, organic solids, and proteins–ligand complexes and has evolved rapidly over the last 4 decades because of advances in force fields, thanks to the development of quantum physics and computational chemistry (Mekni phyto-drugset al., 2019). The simulation is widely used to analyze the structure-to-function relationship of protein and protein–ligand complexes. The current generation molecular dynamics mimic the actual biological systems with a potential simulation period of up to 100 ns for each complex and their behavior in the order of nanoseconds with appropriate system configurations using high-speed supercomputers. It takes thousands to several million steps and involves intra- and interatomic interactions simulated simultaneously, for which supercomputers play a vital role in attaining so. It is essential to study the simulation in the order of shortest duration, preferably femtoseconds, because biomolecules’ structural and functional properties concern nano- and microseconds (Liang et al., 2007).

**FIGURE 5:**
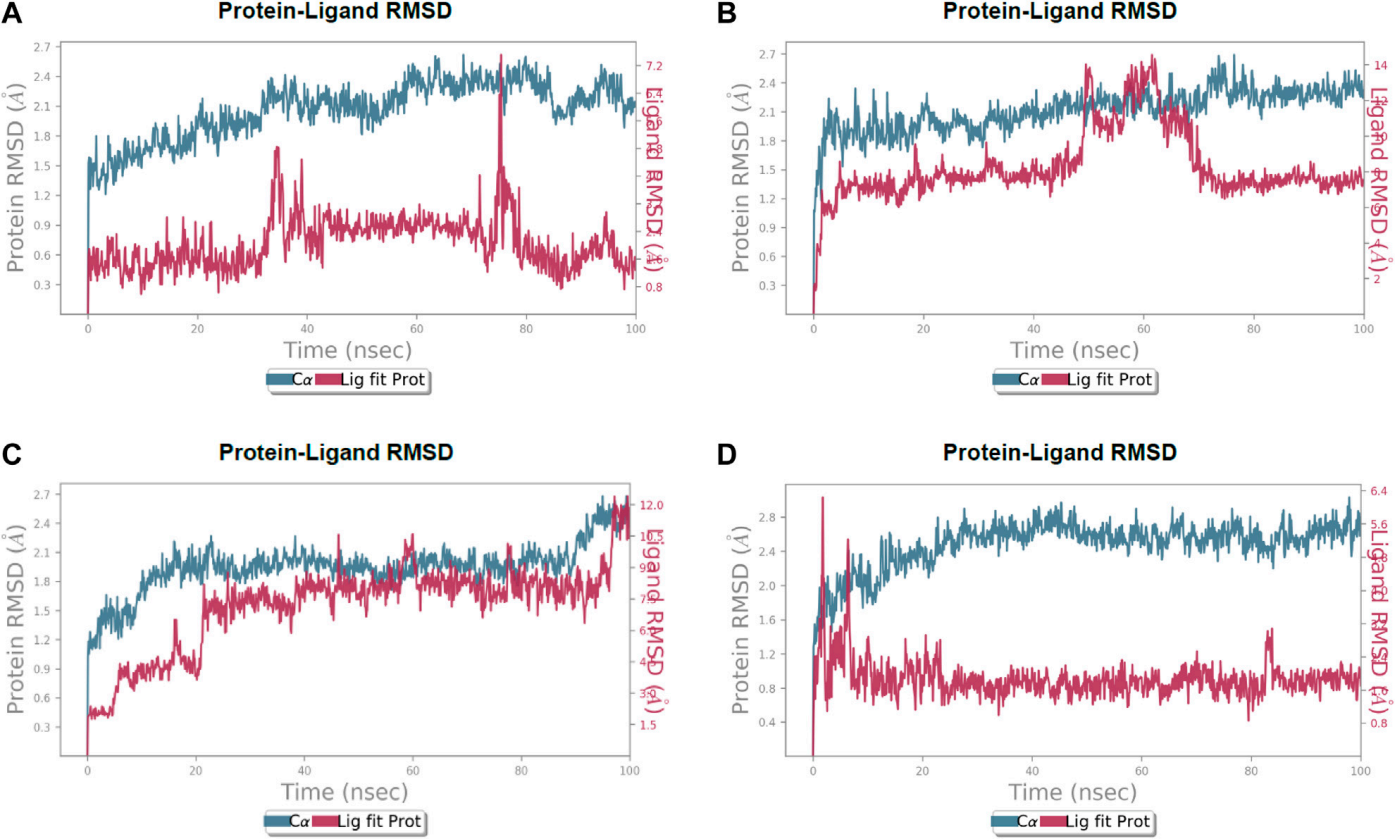
Calculated RMSD values for alpha carbon (Cα) atoms (blue curve) of 3CL protease and protein fit ligands *viz*., **(A)** lochocarpol A, **(B)** broussonol E, **(C)** diplacol, **(D)** dexamethasone, were plotted with respect to 100 ns simulation period.

**FIGURE 6:**
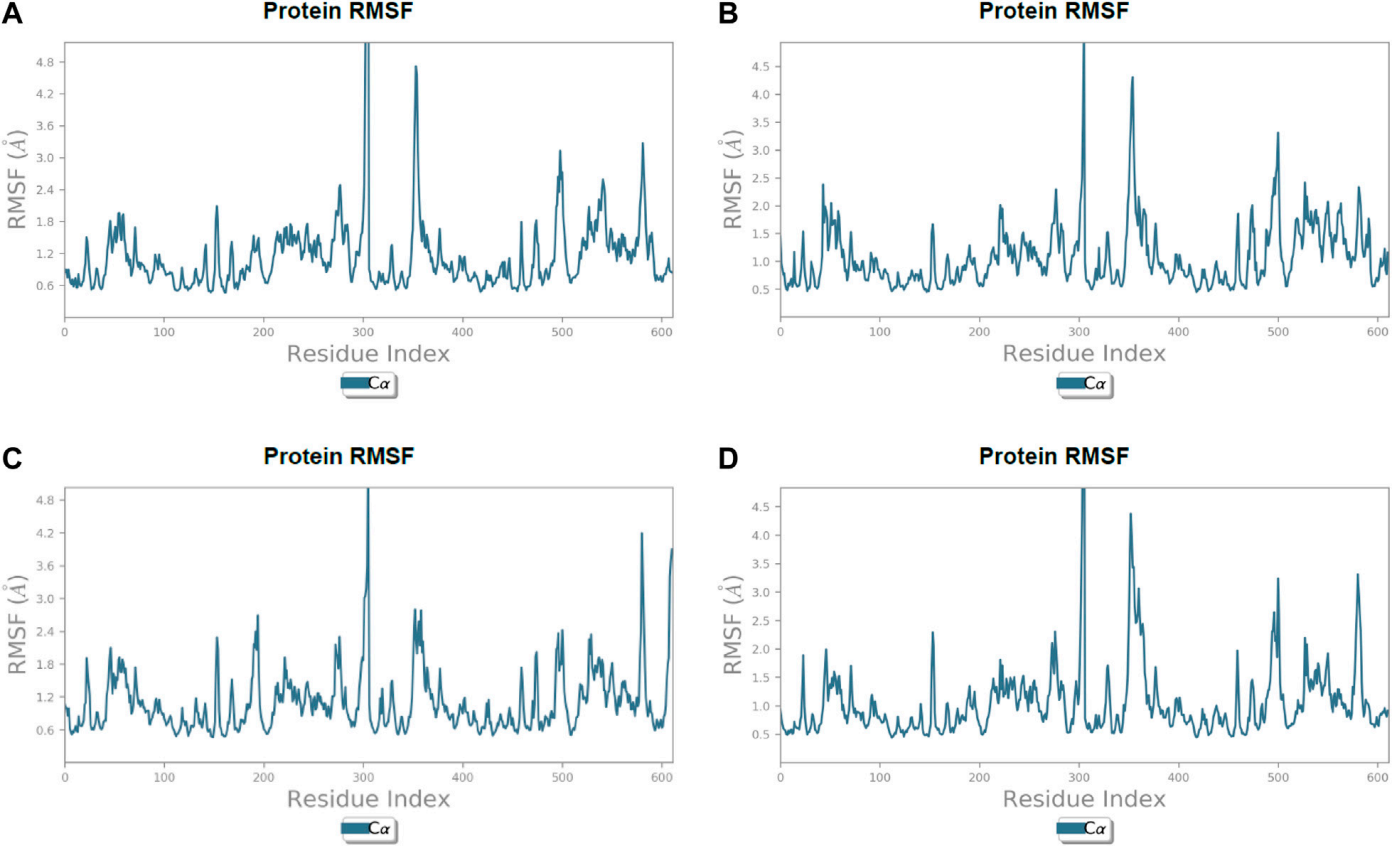
Line representation of the evolution of root-mean-square fluctuation of 3CL protease Cα during the 100 ns MD simulation. **(A)** Lonchocarpol A, **(B)** broussonol E, **(C)** diplacol, **(D)** dexamethasone.

**FIGURE 7:**
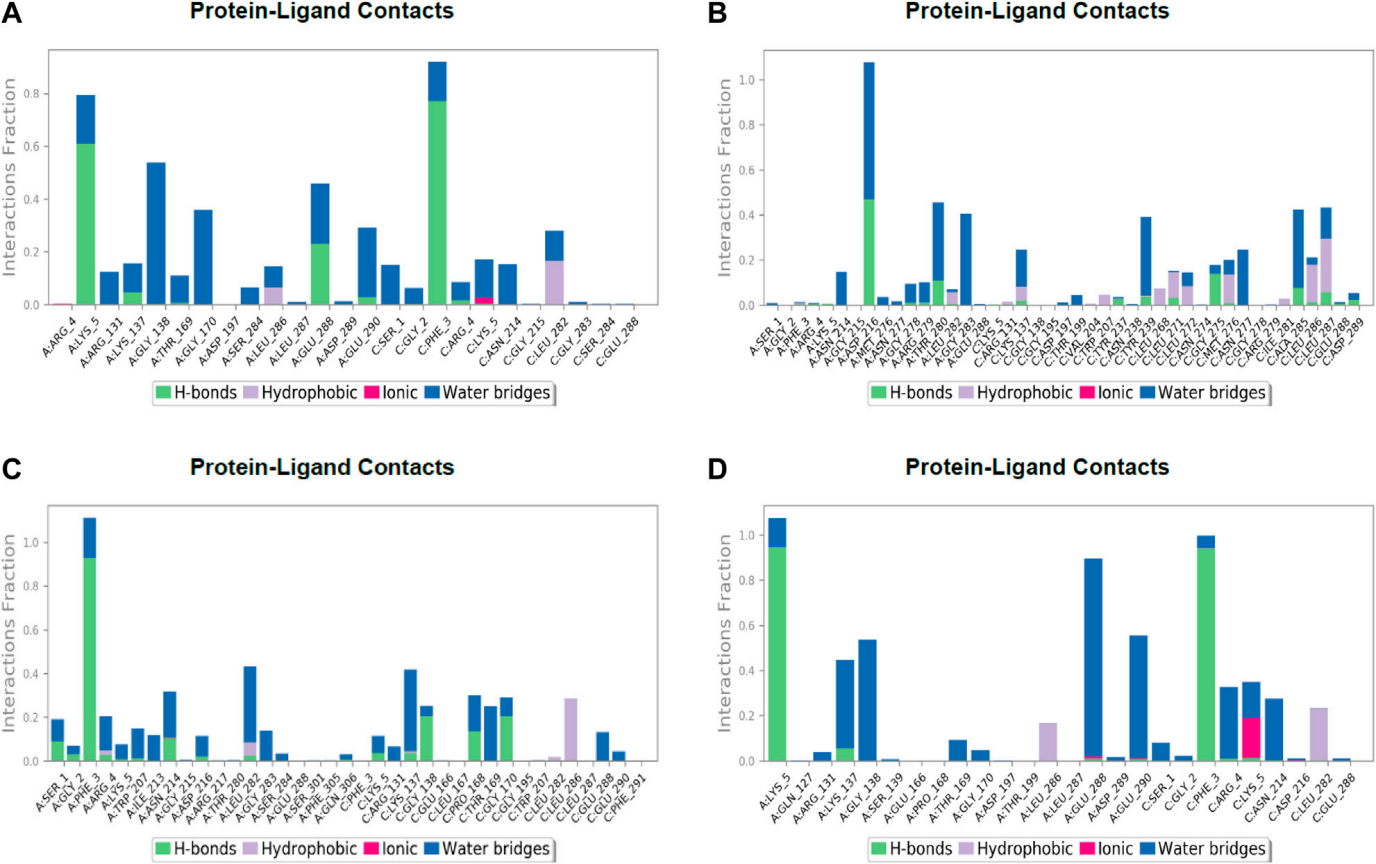
Post simulation analysis of protein–ligand interaction mapping. **(A)** Lonchocarpol **A**–3CLPro complex, **(B)** broussonol **E**–CLPro complex, **(C)** diplacol–3CLPro, **(D)** dexamethasone–3CLPro.

After the chemical profiling, the association of compound complexes was examined, and the dynamic stability of screened compounds was studied using MD simulation at 100 ns in terms of root-mean-square deviation, root-mean-square fluctuations, and molecular contacts (Figures 5–7). This was achieved with the aid of the Desmond module integrated into the Schrodinger suite. Analyzing the molecular dynamics simulation at the atomistic level, all the compounds were relatively stable through the MD simulation period.

Lonchocarpol A-3CLpro complexes were stable within 0–50 ns (Figure 5). However, fluctuations were observed from 55 to 65 ns before the ligand retained its stability. The RMSF analysis explicitly shows that some amino acid residues (PHE3, ARG4, GLY138, and GLU255) contributed to the ligand fluctuations. Clearly, broussonol E was found to be stable when in a complex with the protein backbone, with subtle fluctuation recorded at 25–35 ns and 65–75 ns. The RMSF analysis of broussonol E shows its residue index and established molecular contacts largely dominated by water bridges and hydrophobic interactions. Diplacol demonstrated a varying degree of fluctuations between 0 and 20 ns.

Interestingly, it was found to be very stable from 25 to 100 ns. However, a slight increase in the RMSD value of the ligand was observed toward the end (90–100 ns) of the simulation period, as shown in the trajectory. The reference compound (dexamethasone) peaked at RMSD 2.0 A at 20 ns and established essential interaction profiling such as hydrogen and water bridges. Our results, herein, suggest that the binding of the compounds may prompt conformational alterations. In consistent with this, the analysis of MM-GBSA with trajectory against MM-BGSA without the trajectory residue number showed that the compound complexes showed higher oscillations in backbone residues when compared to other complexes in the systems, as shown in the figure below. This is consistent with the docking results of the four lead compounds that showed the highest binding free energy to other compounds with the low or least binding free energy of −7.3 and −8.1 kcal/mol.

The establishment and immovability of H-bonds were inspected over the simulation period. H-bond features are essential in drug design and discovery due to their irreplaceable role in drug specificity, metabolism, and absorption (Kurczab, 2017). Figure 7 shows that the four lead compounds could establish at least one hydrogen bond with amino acid residues. Thus, the stability of complexes was maintained by H-bond formation with active site residues. According to the docking and MD simulation analyses, the four lead compounds showed good affinity toward COVID-19 compared to the other compounds. However, lonchocarpol A showed a high docking score (−8.644 kcal/mol) and formed pi-stacking interactions with the essential amino acids of the COVID-19 binding domain. The MD simulation of the complexes in the study was very helpful in analyzing the conformational stability and dynamics of the protein and protein–ligand complexes at different nanosecond time intervals, fluctuations, and their deviations from the reference structure on COVID-19.

## 4 Conclusion

This study used an integrated computational approach such as molecular docking, molecular dynamics simulation, and semi-empirical Hamiltonian mechanics to discover flavonoids that could serve as potential therapeutic agent against SARS-CoV-2 therapeutic targets 3CLpro, PLpro, spike glycoprotein, and RdRp. Following the virtual screening, three lead compounds (lonchocarpol A, diplacol, and broussonol E) were identified as novel inhibitors of 3CLpro among the selected targets of SARS-CoV-2 by evaluating binding energy and interaction poses, drug likeness, toxicity, and dynamic stability in comparison to a reference compound (dexamethasone). Molecular docking shows that the compounds have high binding energy, resulting in strong molecular complex formation with the molecular SARS-CoV-2 targets. An atomistic study of the protein–ligand interaction *via* the dynamics simulations shows that deviation in dynamic stability falls within an acceptable range. Therefore, the docked complexes can be considered stable based on intermolecular interactions. The semi-empirical Hamiltonian mechanics elucidated the lead compound polarizability and the high chemical reactivity toward the target receptors. As a result, we propose that the hit compound could serve as a benchmark for developing phytodrugs against COVID-19. However, we recommend further experiments *via in vitro* pharmacological inhibition and neutralization studies to be carried out to validate our claim to develop these compounds as inhibitors of the SARS-CoV-2 therapeutic targets.

## Data Availability

The original contributions presented in the study are included in the article/Supplementary Material; further inquiries can be directed to the corresponding authors.

## References

[B1] Adedeji A. O. Singh K. Calcaterra N. E. DeDiego M. L. Enjuanes L. Weiss S. (2012). Severe acute respiratory syndrome coronavirus replication inhibitor that interferes with the nucleic acid unwinding of the viral helicase. Antimicrob. Agents Chemother. 56 (9), 4718–4728. 10.1128/aac.00957-12 22733076 PMC3421890

[B2] Arjunan V. Mohan S. (2009). Fourier transform infrared and FT-Raman spectra, assignment, *ab initio*, DFT and normal co-ordinate analysis of 2-chloro-4-methyla- niline and 2-chloro-6-methylaniline. Spectrochimica Acta Part A Mol. Biomol. Spectrosc. 72 (2), 436–444. 10.1016/j.saa.2008.10.017 19081287

[B3] Baell J. B. (2016). Feeling nature's PAINS: Natural products, natural product drugs, and Pan assay interference compounds (PAINS). J. Nat. Prod. (Gorakhpur). 79 (3), 616–628. 10.1021/acs.jnatprod.5b00947 26900761

[B4] Baell J. Walters M. A. (2014). Chemistry: Chemical con artists foil drug discovery. Nature 513 (7519), 481–483. 10.1038/513481a 25254460

[B5] Balogun T. A. Ipinloju N. Abdullateef O. T. Moses S. I. Omoboyowa D. A. James A. C. (2021a). Computational evaluation of bioactive compounds from *Colocasia affinis* schott as a novel EGFR inhibitor for cancer treatment. Cancer Inf. 20, 1–12. 10.1177/11769351211049244 PMC8504293 34646061

[B6] Balogun T. A. Iqbal M. N. Saibu O. A. Akintubosun M. O. Lateef O. M. Nneka U. C. (2021b). Discovery of potential HER2 inhibitors from *Mangifera indica* for the treatment of HER2-positive breast cancer: An integrated computational approach. J. Biomol. Struct. Dyn., 1–13. 10.1080/07391102.2021.1975570 34514973

[B7] Benfenati E. Benigni R. Demarini D. M. Helma C. Kirkland D. Martin T. M. (2009). Predictive models for carcinogenicity and mutagenicity: Frameworks, state-of-the-art, and perspectives. J. Environ. Sci. Health Part C 27 (2), 57–90. 10.1080/10590500902885593 19412856

[B8] Chan J. F. Chan K. H. Kao R. Y. To K. K. Zheng B. J. Li C. P. Y. (2013). Broad-spectrum antivirals for the emerging Middle East respiratory syn- drome coronavirus. J. Infect. 67, 606–616. 10.1016/j.jinf.2013.09.029 24096239 PMC7112612

[B9] Chan J. F.-W. Yuan S. Kok K.-H. To K. K.-W. Chu H. Yang J. (2020). A familial cluster of pneumonia associated with the 2019 novel coronavirus indicating person-to-person transmission: A study of a family cluster. Lancet 395 (10223), 514–523. 10.1016/s0140-6736(20)30154-9 31986261 PMC7159286

[B10] Chen Y. Liu Q. Guo D. (2020). Emerging coronaviruses: Genome structure, replication, and pathogenesis. J. Med. Virol. 92, 2249. 10.1002/jmv.26234 32881013 PMC7435528

[B11] Cheng F. Li W. Zhou Y. Shen J. Wu Z. Liu G. (2012). ADMET-SAR: A comprehensive source and free tool for assessment of chemical ADMET properties. J. Chem. Inf. Model. 52, 3099–3105. 10.1021/ci300367a 23092397

[B12] Chukwuemeka P. O. Umar H. I. Iwaloye O. Oretade O. M. Olowosoke C. B. Oretade O. J. (2021). Predictive hybrid paradigm for cytotoxic activity of 1, 3, 4-thiadiazole derivatives as CDK6 inhibitors against human (MCF-7) breast cancer cell line and its structural modifications: Rational for novel cancer therapeutics. J. Biomol. Struct. Dyn., 1–20. 10.1080/07391102.2021.1913231 33890551

[B13] Daina A. Michielin O. Zoete V. (2017). SwissADME: A free web tool to evaluate pharmacokinetics, drug-likeness and medicinal chemistry friendliness of small molecules. Sci. Rep. 7, 42717. 10.1038/srep42717 28256516 PMC5335600

[B14] Darden T. York D. Pedersen L. (1993). Particle mesh Ewald: An*N*⋅log(*N*) method for Ewald sums in large systems. J. Chem. Phys. 98, 10089–10092. 10.1063/1.464397

[B15] de Wilde A. H. Jochmans D. Posthuma C. C. Zevenhoven-Dobbe J. C. van Nieuwkoop S. Bestebroer T. M. (2014). Screening of an FDA-approved compound library identifies four small-molecule inhibitors of Middle East Respiratory syndrome coronavirus replication in cell culture. Antimicrob. Agents Chemother. 14, 4875–4884. 10.1128/aac.03011-14 PMC4136071 24841269

[B16] Dudani T. Saraogi A. (2020). Use of herbal medicines on coronavirus. Act. Scie. Pharma. 11 (6), 61–63. 10.31080/asps.2020.04.0518

[B17] Dutra J. D. L. Filho M. A. M. Rocha G. B. Freire R. O. Simas A. M. Stewart J. J. P. (2013). Sparkle/PM7 lanthanide parameters for the modeling of complexes and materials. J. Chem. Theory Comput. 9, 3333–3341. 10.1021/ct301012h 24163641 PMC3806451

[B18] Dyall J. Coleman C. M. Hart B. J. Venkataraman T. Holbrook M. R. Kindrachuk J. (2014). Repurposing of clinically developed drugs for treatment of Middle East respiratory syndrome coronavirus infection. Antimicrob. Agents Chemother. 58, 4885–4893. 10.1128/aac.03036-14 24841273 PMC4136000

[B19] Friesner R. A. Banks J. L. Murphy R. B. Halgren T. A. Klicic J. J. Mainz D. T. (2004). Glide: A new approach for rapid, accurate docking and scoring. 1. Method and assessment of docking accuracy. J. Med. Chem. 47, 1739–1749. 10.1021/jm0306430 15027865

[B20] Ge X. Y. Li J. L. Yang X. L. Chmura A. A. Zhu G. Epstein J. H. (2013). Isolation and characterization of a bat SARS-like coronavirus that uses the ACE2 receptor. Nature 503, 535–538. 10.1038/nature12711 24172901 PMC5389864

[B21] Genheden S. Ryde U. (2015). The MM/PBSA and MM/GBSA methods to estimate ligand-binding affinities. Expert Opin. Drug Discov. 10, 449–461. 10.1517/17460441.2015.1032936 25835573 PMC4487606

[B22] Greenidge P. A. Kramer C. Mozziconacci J. C. Wolf R. M. (2013). MM/GBSA binding energy prediction on the PDBbind data set: Successes, failures, and directions for further improvement. J. Chem. Inf. Model. 53 (1), 201–209. 10.1021/ci300425v 23268595

[B23] Han D. P. Penn-Nicholson A. Cho M. W. (2006). Identification of critical determinants on ACE2 for SARS-CoV entry and development of a potent entry inhibitor. Virology 350, 15–25. 10.1016/j.virol.2006.01.029 16510163 PMC7111894

[B24] Hanson R. M. (2010). Jmol - a paradigm shift in crystallographic visualization. J. Appl. Crystallogr. 43, 1250–1260. 10.1107/S0021889810030256

[B25] Hanwell M. D. Curtis D. E. Lonie D. C. Vandermeersch T. Zurek E. Hutchison G. R. (2012). Avogadro: An advanced semantic chemical editor, visualization, and analysis platform. J. Cheminform. 4, 17. 10.1186/1758-2946-4-17 22889332 PMC3542060

[B26] Jia Z. Yan L. Ren Z. Wu L. Wang J. Guo J. (2019). Delicate structural coordination of the severe acute respiratory syndrome coronavirus Nsp13 upon ATP hydrolysis. Nucleic Acids Res. 47 (12), 6538–6550. 10.1093/nar/gkz409 31131400 PMC6614802

[B27] Kim H. Y. Eo E. Y. Park H. Kim Y. C. Park S. Shin H. J. (2010). Medicinal herbal extracts of Sophorae radix, Acanthopanacis cortex, Sanguisorbae radix and Torilis fructus inhibit coronavirus replication *in vitro* . Antivir. Ther. 15 (5), 697–709. 10.3851/IMP1615 20710051

[B28] Klamt A. Schuurmann G. (1993). Cosmo: A new approach to dielectric screening in solvents with explicit expressions for the screening energy and its gradient. J. Chem. Soc. Perkin Trans. 2, 799–805. 10.1039/P29930000799

[B29] Kurczab R. (2017). The evaluation of QM/MM-driven molecular docking combined with MM/GBSA calculations as a halogen-bond scoring strategy. Acta Crystallogr. B Struct. Sci. Cryst. Eng. Mat. 73 (2), 188–194. 10.1107/S205252061700138X 28362281

[B30] Li S. W. Wang C. Y. Jou Y. J. Huang S. H. Hsiao L. H. Wan L. (2016). SARS coronavirus papain-like protease inhibits the TLR7 signaling pathway through removing Lys63-linked polyubiquitination of TRAF3 and TRAF6. Int. J. Mol. Sci. 17, 678. 10.3390/ijms17050678 PMC4881504 27164085

[B31] Li W. Moore M. J. Vasilieva N. Sui J. Wong S. K. Berne M. A. (2003). Angiotensin-converting enzyme 2 is a functional receptor for the SARS coronavirus. Nature 426, 450–454. 10.1038/nature02145 14647384 PMC7095016

[B32] Liang Z. Liu F. Grundke-Iqbal I. Iqbal K. Gong C.-X. (2007). Downregulation of cAMP-dependent protein kinase by over-activated calpain in Alzheimer disease brain. J. Neurochem. 103 (6), 2462–2470. 10.1111/j.1471-4159.2007.04942.x 17908236 PMC2262109

[B33] Ling C. (2020). Traditional Chinese medicine is a resource for drug discovery against 2019 novel coronavirus (SARS-CoV-2). J. Integr. Med. 18 (2), 87–88. 10.1016/j.joim.2020.02.004 32122812 PMC7129043

[B34] Lipinski C. A. Lombardo F. Dominy B. W. Feeney P. J. (2001). Experimental and computational approaches to estimate solubility and permeability in drug discovery and development settings. Adv. Drug Deliv. Rev. 46, 3–25. 10.1016/s0169-409x(96)00423-1 11259830

[B35] Liu C. Zhou Q. Li Y. Garner L. V. Watkins S. P. Carter L. J. (2020). Research and development on therapeutic agents and vaccines for COVID-19 and related human coronavirus diseases. ACS Cent. Sci. 6, 315–331. 10.1021/acscentsci.0c00272 32226821 PMC10467574

[B36] Luk H. K. H. Li X. Fung J. Lau S. K. P. Woo P. C. Y. (2019). Molecular epidemiology, evolution and phylogeny of SARS coronavirus. Infect. Genet. Evol. 71, 21–30. 10.1016/j.meegid.2019.03.001 30844511 PMC7106202

[B37] Maragatham G. Selvarani S. Rajakumar P. Lakshmi S. (2019). Structure determination and quantum chemical analysis of chalcone derivatives. J. Mol. Struct. 1179, 568–575. 10.1016/j.molstruc.2018.11.048

[B38] Mekni N. De Rosa M. Cipollina C. Gulotta M. R. De Simone G. Lombino J. (2019). *In silico* insights towards the identification of NLRP3 druggable hot spots. Int. J. Mol. Sci. 20 (20), 4974. 10.3390/ijms20204974 PMC6834175 31600880

[B39] Millet J. K. Whittaker G. R. (2015). Host cell proteases: Critical determinants of coronavirus tropism and pathogenesis. Virus Res. 202, 120–134. 10.1016/j.virusres.2014.11.021 25445340 PMC4465284

[B40] Muhammad S. Lai C-H. Al-Sehemi A. G. Alshahrani T. Iqbal J. Ayub K. (2021). Exploring the twisted molecular configurations for tuning their optical and nonlinear optical response properties: A quantum chemical approach. J. Mol. Graph. Model. 102, 107766. 10.1016/j.jmgm.2020.107766 33069123

[B41] Muthu S. Ramachandran G. Paulraj R. I. Swaminathan T. (2014). Quantum mechanical study of the structure and spectroscopic (FTIR, FT-Raman), first-order hyperpolarizability and NBO analysis of 1, 2-benzoxazol-3-ylmethane sulfonamide. Spectrochim. Acta Part A Mol. Biomol. Spectrosc. 128, 603–613. 10.1016/j.saa.2014.02.183 24691375

[B42] Omrani A. S. Saad M. M. Baig K. Bahloul A. Abdul-Matin M. Alaidaroos A. Y. (2014). Ribavirin and interferon alfa-2a for severe Middle East respiratory syndrome coronavirus infection: A retrospec- tive cohort study. Lancet Infect. Dis. 14, 1090–1095. 10.1016/s1473-3099(14)70920-x 25278221 PMC7106357

[B43] Parrinello M. Rahman A. (1981). Polymorphic transitions in single crystals: A new molecular dynamics method. J. Appl. Phys. 52, 7182–7190. 10.1063/1.328693

[B44] Pearson R. G. (1986). Absolute electronegativity and hardness correlated with molecular orbital theory. Proc. Natl. Acad. Sci. U. S. A. 83, 8440–8441. 10.1073/pnas.83.22.8440 16578791 PMC386945

[B45] Pearson R. G. (1990). Hard and soft acids and bases-the evolution of a chemical concept. Coord. Chem. Rev. 100, 403–425. 10.1016/0010-8545(90)85016-l

[B46] Phillips J. C. (1961). Generalized koopmans’ theorem. Phys. Rev. 123, 420–424. 10.1103/PhysRev.123.420

[B47] Pillaiyar T. Manickam M. Namasivayam V. Hayashi Y. Jung S. H. (2016). An overview of severe acute respiratory syndrome-coronavirus (SARSCoV) 3CL protease inhibitors: Peptidomimetics and small molecule chemotherapy. J. Med. Chem. 59, 6595–6628. 10.1021/acs.jmedchem.5b01461 26878082 PMC7075650

[B48] Ruan Z. Liu C. Guo Y. He Z. Huang X. Jia X. (2020). SARS-CoV-2 and SARS-CoV: Virtual screening of potential inhibitors targeting RNA-dependent RNA polymerase activity (NSP12). J. Med. Virol. 93, 389–400. 10.1002/jmv.26222 32579254 PMC7361265

[B49] Salvatore M. J. King A. B. Graham A. C. Onishi H. R. Bartizal K. F. Abruzzo G. K. (1998). Antibacterial activity of lonchocarpol A. J. Nat. Prod. (Gorakhpur). 61 (5), 640–642. 10.1021/np9703961 9599265

[B50] Sastry G. M. Adzhigirey M. Day T. Annabhimoju R. Sherman W. (2013). Protein and ligand preparation: Parameters, protocols, and influence on virtual screening enrichments. J. Comput. Aided. Mol. Des. 27, 221–234. 10.1007/s10822-013-9644-8 23579614

[B51] Schrödinger (2018). Schrödinger release 201 8-4: Maestro schrödinger. New York, NY: LLC.

[B52] Schrödinger (2020). Schrödinger release 2020–2: Prime, schrödinger. New York, NY: LLC.

[B53] Seah I. Agrawal R. (2020). Can the coronavirus disease 2019 (COVID-19) affect the eyes? A review of coronaviruses and ocular implications in humans and animals. Ocul. Immunol. Inflamm. 28, 391–395. 10.1080/09273948.2020.1738501 32175797 PMC7103678

[B54] Stewart J. J. P. (1990). MOPAC: A semiempirical molecular orbital program. J. Comput. Aided. Mol. Des. 4, 1–103. 10.1007/BF00128336 2197373

[B55] Sylaja B. Gunasekaran S. Srinivasan S. (2017). Vibrational, NLO, NBO, NMR, frontier molecular orbital and molecular docking studies of diazepam. Mater. Res. Innovations 22, 1–13. 10.1080/14328917.2017.1324356

[B56] Tripathi S. K. Muttineni R. Singh S. K. (2013). Extra precision docking, free energy calculation and molecular dynamics simulation studies of CDK2 inhibitors. J. Theor. Biol. 334, 87–100. 10.1016/j.jtbi.2013.05.014 23727278

[B57] Vellingiri B. Jayaramayya K. Iyer M. Narayanasamy A. Govindasamy V. Giridharan B. (2020). COVID-19: A promising cure for the global panic. Sci. Total Environ. 725, 138277. 10.1016/j.scitotenv.2020.138277 32278175 PMC7128376

[B58] Wang S. Q. Du Q. S. Zhao K. Li A. X. Wei D. Q. Chou K. C. (2007). Virtual screening for finding natural inhibitor against cathepsin-L for SARS therapy. Amino Acids 33 (1), 129–135. 10.1007/s00726-006-0403-1 16998715 PMC7087620

[B59] World Health Organization (2004). SARS: Clinical trials on treatment using a combination of traditional Chinese medicine and western medicine. Beijing: World Health Organization.

[B60] Wu C. Y. Jan J. T. Ma S. H. Kuo C. J. Juan H. F. Cheng Y. S. E. (2004). Small molecules targeting severe acute respiratory syndrome human coronavirus. Proc. Natl. Acad. Sci. U. S. A. 101 (27), 10012–10017. 10.1073/pnas.0403596101 15226499 PMC454157

[B61] Xu C. Ke Z. Liu C. Wang Z. Liu D. Zhang L. (2020). Systemic *in silico* screening in drug discovery for Coronavirus Disease (COVID-19) with an online interactive web server. J. Chem. Inf. Model. 60, 5735–5745. 10.1021/acs.jcim.0c00821 32786695

[B62] Yu M.-S. Lee J. Lee J. M. Kim Y. Chin Y.-W. Jee J.-G. (2012). Identification of myricetin and scutellarein as novel chemical inhibitors of the SARS coronavirus helicase, NsP13. Bioorg. Med. Chem. Lett. 22 (12), 4049–4054. 10.1016/j.bmcl.2012.04.081 22578462 PMC7127438

[B63] Zumla A. Chan J. F. Azhar E. I. Hui D. S. Yuen K. Y. (2016). Corona viruses-drug discovery and therapeutic options. Nat. Rev. Drug Discov. 15, 327–347. 10.1038/nrd.2015.37 26868298 PMC7097181

